# Early Gnathostome Phylogeny Revisited: Multiple Method Consensus

**DOI:** 10.1371/journal.pone.0163157

**Published:** 2016-09-20

**Authors:** Tuo Qiao, Benedict King, John A. Long, Per E. Ahlberg, Min Zhu

**Affiliations:** 1 Key Laboratory of Vertebrate Evolution and Human Origins of Chinese Academy of Sciences, Institute of Vertebrate Paleontology and Paleoanthropology, Chinese Academy of Sciences, Beijing, China; 2 School of Biological Sciences, Flinders University, Adelaide, South Australia, Australia; 3 Department of Organismal Biology, Evolutionary Biology Centre, Uppsala University, Norbyvägen, Uppsala, Sweden; Laboratoire Arago, FRANCE

## Abstract

A series of recent studies recovered consistent phylogenetic scenarios of jawed vertebrates, such as the paraphyly of placoderms with respect to crown gnathostomes, and antiarchs as the sister group of all other jawed vertebrates. However, some of the phylogenetic relationships within the group have remained controversial, such as the positions of *Entelognathus*, ptyctodontids, and the *Guiyu-*lineage that comprises *Guiyu*, *Psarolepis* and *Achoania*. The revision of the dataset in a recent study reveals a modified phylogenetic hypothesis, which shows that some of these phylogenetic conflicts were sourced from a few inadvertent miscodings. The interrelationships of early gnathostomes are addressed based on a combined new dataset with 103 taxa and 335 characters, which is the most comprehensive morphological dataset constructed to date. This dataset is investigated in a phylogenetic context using maximum parsimony (MP), Bayesian inference (BI) and maximum likelihood (ML) approaches in an attempt to explore the consensus and incongruence between the hypotheses of early gnathostome interrelationships recovered from different methods. Our findings consistently corroborate the paraphyly of placoderms, all ‘acanthodians’ as a paraphyletic stem group of chondrichthyans, *Entelognathus* as a stem gnathostome, and the *Guiyu*-lineage as stem sarcopterygians. The incongruence using different methods is less significant than the consensus, and mainly relates to the positions of the placoderm *Wuttagoonaspis*, the stem chondrichthyan *Ramirosuarezia*, and the stem osteichthyan *Lophosteus*—the taxa that are either poorly known or highly specialized in character complement. Given that the different performances of each phylogenetic approach, our study provides an empirical case that the multiple phylogenetic analyses of morphological data are mutually complementary rather than redundant.

## Introduction

Jawed vertebrates or gnathostomes comprise 99.8% of living vertebrate species [1]. Paleozoic jawed vertebrates are divided into four broad categories: chondrichthyans and osteichthyans, both with extant representatives, and two entirely extinct assemblages, acanthodians and placoderms [2–4].

Chondrichthyans lack dermal bones and are characterised by an endoskeleton of eventually calcified cartilage. They are represented by the modern sharks and rays (Elasmobranchii) and chimaerids (Holocephali). Fossil remains of chondrichthyans, apart from isolated teeth and fin spines, are rare and the anatomy of early chondrichthyans (Early Devonian or older) is known only from one isolated braincase [5] and one nearly complete specimen, *Doliodus* [6]. Osteichthyans are characterized by endochondral bone within their endoskeleton, and include about 98% of extant vertebrate species. Osteichthyans diverged along two major lineages, namely actinopterygians (bichirs, sturgeons, gars, bowfins and teleosts) and sarcopterygians (coelacanths, lungfishes and tetrapods). ‘Acanthodians’ are a group of jawed vertebrates with small, square-crowned scales, spines before the dorsal, anal and paired fins, and a heterocercal caudal fin. This exclusively Paleozoic group exhibits a mosaic of shark- and bony fish-like characters that has long given them prominence in discussions of early gnathostome evolution [2, 4, 7–11]. Their relationships with modern gnathostomes have remained mysterious, partly because the detailed endoskeletal structure is only known by the latest, highly specialized *Acanthodes bronni* [7, 8, 10, 12–15]. Placoderms, with their characteristic armor of bony plates, were the most successful and diverse group of jawed fishes during the Late Silurian and Devonian. They have an excellent fossil record because their dermal bones were generally robust and easily preserved. Placoderms are of great significance as a model for the ancestral gnathostome condition.

The phylogeny of early gnathostomes is one of the puzzling issues in the study of early vertebrates. Our understanding of early gnathostomes has improved greatly in recent years as a result of new discoveries [16–20] and the re-examinations of available fossils [10, 11, 21]. Although some areas have remained controversial, such as the interrelationships of placoderms, recent studies recovered consistent phylogenetic scenarios of early gnathostomes, such as the paraphyly of placoderms, and antiarchs as the sister to all other jawed vertebrates [4, 9, 18, 20–22].

### Conflicting Phylogenies of Early Gnathostomes

**Fig 1:**
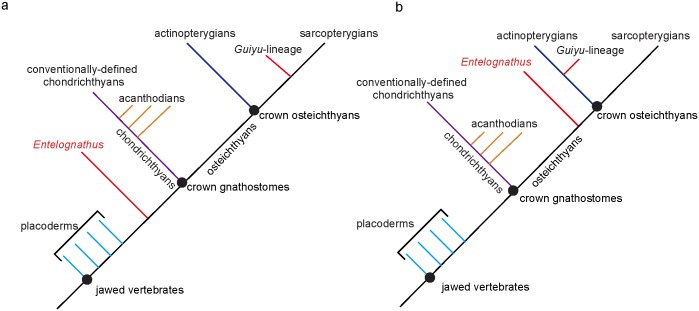
Summary trees of conflicts between Zhu et al.’s [18] (a) and Long et al.’s [20] (b).

Recently, Long et al. [20] applied maximum parsimony (MP) analysis to the dataset of Dupret et al. [21] with the addition of four extra characters and 14 additional placoderm taxa, but recovered different results from other phylogenies [18, 22] in the positions of *Entelognathus*, ptyctodontids and the *Guiyu*-lineage (*Guiyu*, *Psarolepis* and *Achoania*) (Fig 1).

*Entelognathus* was discovered from the Late Silurian in China. It combines typical placoderm characters of dermal skeleton and braincase with osteichthyan-like marginal jaw bones, and has been considered in a polytomy with arthrodires, ptyctodontids and crown gnathostomes or as the sister group of crown gnathostomes [18, 21]. Long et al. [20] recovered *Entelognathus* as a stem osteichthyan. However, they have admitted that “this position on the tree could be an artefact” caused by the absence of dermal bone jaw characters for chondrichthyans and acanthodians. Furthermore, they stated “the braincase and palatoquadrate of this taxon clearly distinguish it from the Osteichthyes” ([20]: supplementary information). This new position impacts our understanding of various character acquisitions and character polarities related to the origins of gnathostomes, such as the innovation of marginal jaw bones (premaxilla, maxilla and dentary) and operculogular series. For example, under the recently resolved framework in which all acanthodians are placed in the stem group of chondrichthyans [18, 20, 22], given *Entelognathus* as the sister group of crown gnathostomes, the marginal jaw bones and operculogular series are possibly present in the common ancestor of chondrichthyans and osteichthyans, and thus might have been secondarily lost in acanthodians and chondrichthyans. However, if we accept *Entelognathus* as a stem osteichthyan as in Long et al. [20], the marginal jaw bones and operculogular series are regarded as synapomorphies of osteichthyans.

Three members of the *Guiyu* lineage are all found from the Late Silurian—Early Devonian of South China and northern Vietnam [16, 23–25]. *Guiyu* and *Psarolepis* manifest a combination of features found in both sarcopterygians and actinopterygians (e.g. pectoral girdle structures, the cheek and operculo-gular bone pattern, and scale articulation) [16, 24]. They also reveal a combination of osteichthyan and non-osteichthyan features, including spine-bearing pectoral girdles and spine-bearing median dorsal plates found in non-osteichthyan gnathostomes, as well as cranial morphology and derived macromeric squamation found in crown osteichthyans [16]. They were referred to stem sarcopterygians in most earlier studies [16, 26, 27]. The phylogenetic analysis in Zhu et al. [24] assigned two possible positions for *Psarolepis*, either as a stem sarcopterygian or as a stem osteichthyan. Qu et al. [28] reported that the absence of tooth enamel in *Psarolepis*, contrasting with its presence in both actinoptergians and sarcopterygians. This character distribution corroborates *Psarolepis* as a stem osteichthyan. The phylogenetic hypothesis proposed by Long et al. [20] focused on placoderms and did not add many actinopterygian and sarcopterygian taxa in their analysis, thus they placed the *Guiyu*-lineage among stem actinopterygians. This unexpected placement, which was followed by Schultze [29], poses a challenge to attempts at polarizing the plesiomorphic osteichthyan and sarcopterygian characters and reconstructing osteichthyan morphotype.

The position of ptyctodontids is another major discrepancy between the phylogenies of Zhu et al. [18] and Long et al. [20]. Miles and Young [30] proposed ptyctodontids as the sister group of all other placoderms. Goujet [31], and Goujet and Young [32], later set ptyctodontids as the sister group of petalichthyids, and Long et al. [20] placed ptyctodontids within a paraphyletic Petalichthyida. By comparison, Zhu et al. [18] assigned ptyctodontids crownward to the petalichthyid *Macropetalichthys*, although the relationships of ptyctodontids, arthrodires, *Entelognathus* and crown gnathostomes were unresolved as a polytomy.

To find the sources of these conflicting phylogenies, we re-scrutinized the datasets of Dupret et al. [21] and Long et al. [20], which were in turn modified from Zhu et al. [18], and found that some states in the former were erroneously coded from Zhu et al. [18] by an inadvertent copy (see the Materials and Methods). These confused codings have a significant effect on the phylogenetic results, especially for the positions of *Entelognathus* and the *Guiyu* lineage. The clarification of this problem will help to evaluate the phylogeny of Long et al. [20]. Based on the revised dataset of Long et al. [20], we revisited the phylogeny of early gnathostomes and further expanded the dataset from other recent works by Giles et al. [22], Brazeau and de Winter [11] and Lu et al. [33].

For a long time, the MP method had been employed in most phylogenetic studies of paleontological morphological data. Along with their extensive adoption in molecular phylogenetic studies, probability-based methods have been used more often in palaeontological phylogenetic analyses recently [34]. The probability-based methods differ from the MP method mainly in each branch of the recovered tree having a given ‘length’ that is equally applied to all characters in order to determine the probability of change along that branch [34–36]. Due to these different principles, the positions of some taxa vary across the trees recovered from different phylogenetic methods. To evaluate the robustness and confidence of the analysed data, we conducted two types of probabilistic analysis: maximum likelihood (ML) and Bayesian inference (BI) analyses to the expanded dataset, in addition to the MP method.

## Materials and Methods

The character data entry and formatting were performed in Mesquite (version 2.5) [37]. All characters were unordered, with the exception of two multistate characters which had been ordered as they were first defined (Characters 294 and 302).

### Maximum Parsimony Analysis

The dataset was subjected to the MP analysis in TNT software package [38]. The analyses were conducted using a traditional search strategy, with default settings apart from the following: 10,000 maximum trees in memory and 1,000 replications. Bremer support values were generated in TNT by applying the ‘New Traditional Search’ using TBR and collecting suboptimal topologies with 1,000 replicates. Bootstrap values were generated in TNT using 1,000 replicates. We used the "unambiguous changes only" option in WinClada [39] to optimize character state changes on the cladograms.

### Bayesian Inference Analysis

The BI analyses were conducted using MrBayes 3.1.2 [40, 41]. Osteostracans were set as the outgroup, and the coding showing polymorphisms were changed to ‘?’. The analyses used the Lewis stochastic model [42] for standard datatype with coding set to ‘variable’ and a gamma parameter to account for rate variation across traits. Four simultaneous analyses were run, each including four independent Markov chains: one cold chain and three incrementally heated chains. The analysis was run for 1×10^7^ generations. Samples were taken every 1×10^3^ generations, resulting in a total of 1×10^4^ samples for each of the parallel analyses. Convergence of all four runs was confirmed by superposition of likelihood and posterior probability traces, as viewed in Tracer [43], and effective sample size >1,000 for every parameter. Standard deviation of split frequencies was approximately 0.005 across the four runs. Convergence of the MCMC was graphically checked by plotting frequencies of splits found in Bayesian run 1 against frequencies found in the other three runs, using the online tool AWTY [44]. The first 1.0×10^3^ samples for each run, representing the ‘burn-in’ period, were discarded. The 50% majority-rule consensus tree was computed for the sampled generations of one analysis.

### Maximum Likelihood Analysis

The ML analyses were conducted using RAxML v.8.2.4 [45], with 1,000 bootstraps followed by a maximum likelihood search. Commands used were ‘-f a -m MULTIGAMMA -K MK -#1,000’. The 50% majority-rule consensus tree was computed for the 1,000 bootstrap trees. ML analysis used the Lewis stochastic model [42] with a gamma parameter to account for rate variation across traits.

### Revision of Long et al.’s [20] Dataset

The following are the revisions of seven characters, which were erroneously coded in Dupret et al.’s [21] dataset by an inadvertent copy from Zhu et al. [18] and then adopted by Long et al. [20]. Other revisions see the supplementary materials online.

Character 128–Fin spines with ridges (Character 132 in [18]): Zhu et al. [18] had a refined formulation for this character, and restricted the character to pectoral fin spines. Following Brazeau (2009, Character 125) and Davis et al. (2012, Character 128), the spinal plates of placoderms were treated as homologues of pectoral fin spines of acanthodians and chondrichthyans. Accordingly, the codings in many placoderm taxa, *Guiyu* and *Psarolepis* were changed back to “1” (presence). Galeaspids were coded as “-” (inapplicable), following Zhu et al. [18].

Character 144–Type of dermal neck-joint (Character 169 in [18]): *Parayunnanolepis* was coded as “2” (reversed ginglymoid).

Character 155–Shape of parasphenoid denticulated field (Character 240 in [18]): Zhu et al. [18] identified three states for this character. By oversight, Dupret et al. [21] mixed states 1 and 2 of this character as a single state, and the third state (slender, splint-shaped parasphenoid, state 2 in [18]) was not coded. The formulation and state codings of this character were restored from Zhu et al. [18].

Character 157–Resorption and redeposition of odontodes (Character 139 in [18]): Dupret et al. [21] missed the codings of the second state (developed resorption and redeposition of odontodes) in their dataset, thus rendered this character uninformative. The codings of the second state were restored from Zhu et al. [18].

Character 177–Dermal neck-joint between paired main-lateral-line-bearing bones of skull and shoulder girdle (Character 168 in [18]): *Macropetalichthys* and *Romundina* were coded as “1” (presence).

Characters 188–Course of ethmoid commissure (Character 183 in [18]) and Character 211–Parasymphysial plate (Character 209 in [18]): Zhu et al. [18] identified three states for each character. By oversight, Dupret et al. [21] mixed states 0 and 1 of each character as a single state, and rendered their codings confused. The formulation and state codings of these two characters were restored from Zhu et al. [18].

### Expanded Dataset Combined from Long et al. [20] and other recent works

Recently, some new datasets of early gnathostomes have been explored for phylogenetic analysis [11, 22, 33]. Here we expanded the dataset of Long et al. [22] with the addition of 84 characters from Giles et al. [22], Brazeau and de Winter [11] and Lu et al. [33]. We have used characters from these four datasets to keep the analyses as comparable as possible. The nomenclature of the four pairs of transverse cranial processes (i.e. postorbital process, transverse otic process, craniospinal process and vagal process) proposed by Giles et al. [22] is adopted herein and six characters (Characters 76, 93, 94, 224, 230, 232) relating to these processes used by Long et al. [20] were deleted to avoid duplication. Character 6 of Long et al. [20] relating to cosmine is represented by four characters (Characters 5–8) in Giles et al.’s [22] dataset, thus this character is also deleted. The expanded dataset includes 335 characters: 252 characters adopted from Long et al. ‘s [20] dataset (Characters 1–252), 71 ones taken from Giles et al.’s [22] (Characters 253–323), one from Brazeau and de Winter’s [11] (Characters 324), and 11 from Lu et al.’s [33] (Characters 325–335).

12 taxa (*Yunnanolepis* [46–48], *Eurycaraspis* [49], *Kujdanowiaspis* [50, 51], *Jagorina* [50], *Gemuendina* [52, 53], *Janusiscus* [22], *Ramirosuarezia* [54], *Gyracanthides* [13, 55, 56], *Latviacanthus* [57], *Helodus* [58], *Kentuckia* [59], *Glyptolepis* [60–63]) have been newly added based on the datasets of Giles et al. [22] and Brazeau and de Winter [11]. Thus, the former 252 characters taken from Long et al. [20] for these taxa were newly coded. 25 taxa (*Sinolepis* [64, 65], *Microbrachius* [20, 66], *Remigolepis* [67–70], *Diandongpetalichthys* [71, 72], *Quasipetalichthys* [73], *Wuttagoonaspis* [74, 75], *Groenlandaspis* [76, 77], *Sigaspis* [78], *Dicksonosteus* [31, 79], *Parabuchanosteus* [20, 80], *Holonema* [67, 81, 82], *Compagopiscis* [82, 83], *Materpiscis* [84], *Rhadinacanthus* [13, 85], *Vernicomacanthus* [13], *Lophosteus* [86–91], *Osorioichthys* [92], *Meemannia* [33, 93, 94], *Achoania* [25, 95, 96], *Miguashaia* [97–100], *Styloichthys* [95, 101], *Youngolepis* [102–106], *Powichthys* [107–110], *Kenichthys* [111, 112], and *Osteolepis* [90, 113, 114]) only appeared in Long et al.’s [20] dataset and the latter 84 characters taken from other datasets [11, 22, 33] for these taxa were newly coded. The extended dataset uses 2 jawless taxa: Osteostraci [115–117] and Galeaspida [115, 118–121] as outgroups. 101 ingroup taxa include 34 placoderms, 25 acanthodians, 14 conventionally-defined chondrichthyans, 25 osteichthyans, *Entelognathus* [18], *Janusiscus* [22] and *Ramirosuarezia* [54].

## Results and Discussion

### Comparison between the Phylogeny based on the Revised Dataset and the Phylogeny of Long et al. [20]

The MP analysis used the revised dataset with the exclusion of six placoderms (*Sinolepis*, *Gavinaspis*, *Sigaspis Parayunnanolepis*, *Quasipetalichthys*, and *Diandongpetalichthys*) with >75% missing data as in the earlier version of this analysis [20]. If only the seven erroneously-coded characters by inadvertent copy are revised, *Entelognathus* falls into a polytomy with osteichthyans and chondrichthyans in the strict consensus tree (SCT) and is recovered as the sister taxon of all crown gnathostomes in the 50% majority-rule consensus tree (MCT), consistent with the position as it was first described [18] (S1b Fig). Therefore, the six erroneously-coded characters affect the phylogenetic position of *Entelognathus*, but the *Guiyu*-lineage is still close to actinopterygians.

**Fig 2:**
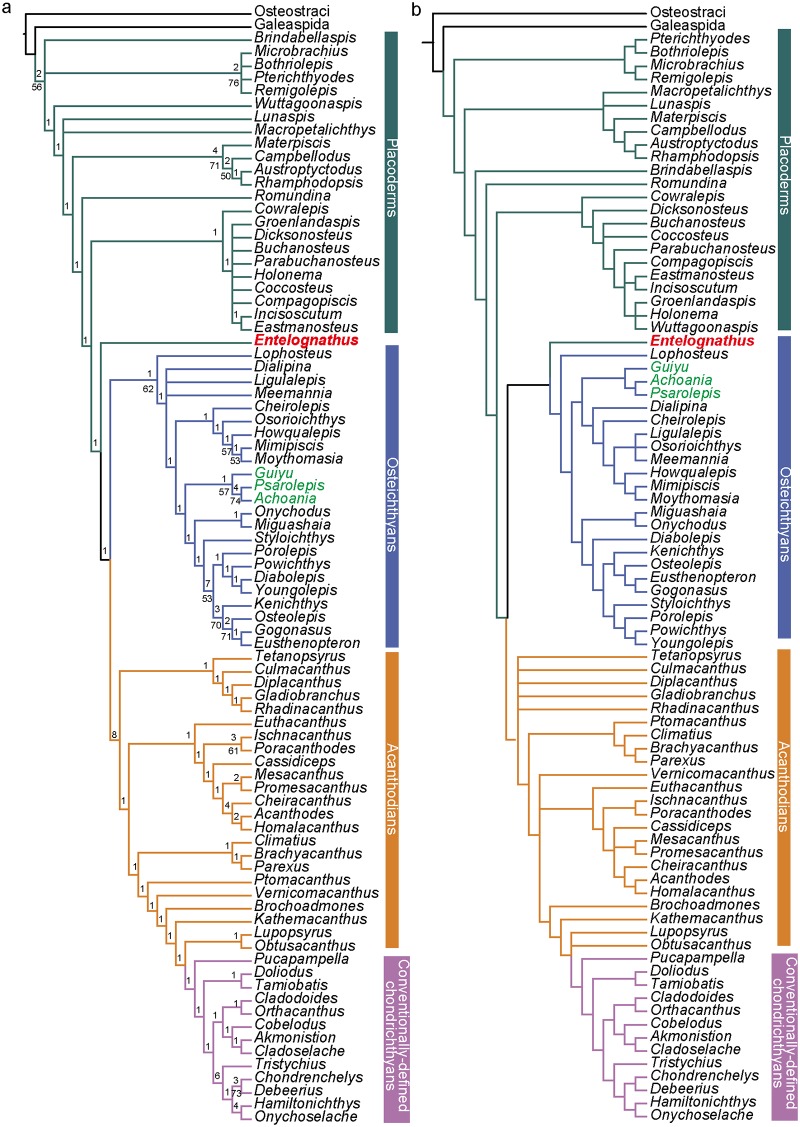
(a) The strict consensus tree of 36 most parsimonious trees based on the dataset revised from Long et al. [20] (85 taxa). (b) the strict consensus tree based on the original dataset of Long et al. [20]. Numbers on branches denote Bremer and bootstrap support.

After all revisions were made, the analysis produced 36 most parsimonious trees (MPT) of 650 steps [consistency index (CI) = 0.4231; homoplasy index (HI) = 0.5769; retention index (RI) = 0.8250; rescaled consistency index (RCI) = 0.3490]. These trees are summarized as a strict consensus tree (SCT) (Fig 2a) and a 50% majority-rule consensus tree (MCT) (S2 Fig). One MPT, which agrees well with the 50% MCT, is selected for illustrating inferred character transformations at various nodes (S3 Fig).

The SCT makes a few departures from the original result of Long et al. [20] (Fig 2b). *Brindabellaspis* and antiarchs are placed in a polytomy with other gnathostomes. *Wuttagoonaspis* is resolved as the sister taxon to all other members in a major clade grouping the remaining gnathostomes except *Brindabellaspis* and antiarchs. By comparison, *Wuttagoonaspis* is placed in the arthrodire clade in the original SCT (Fig 2b).

The grouping of petalichthyids and ptyctodontids is not supported as in the original analysis. *Lunaspis* and *Macropetalichthys* fall into a polytomy with the remaining gnathostomes except *Brindabellaspis*, antiarchs and *Wuttagoonaspis*.

*Entelognathus* is recovered as the sister taxon of all crown gnathostomes, consistent with the position as it was first described [18]. The *Guiyu* lineage is placed as stem sarcopterygians rather than stem actinopterygians as in the phylogeny of Long et al. [20]. All sampled acanthodians are placed as stem chondrichthyans (*contra* [9] and [10]), but as a paraphyletic assemblage [18, 20]. Dupret et al. [21] placed acanthodians as the monophyletic sister group to chondrichthyans. However, that analysis was incorrect as pointed out by Long et al. [20]. The real results also support the acanthodians as a paraphyletic assemblage ([20]: extended data figure 7).

The original and revised matrices with all 91 taxa (i.e. without deleting those six taxa with >75% missing data) were also analyzed using the MP method. The SCT (S4 Fig) recovered from the original dataset shows that *Entelognathus* is a stem gnathostome and the *Guiyu* lineage is close to sarcopterygians. Thus, the deletion of the six taxa seems to affect the topology a lot. After revision, 532 MPTs of 662 steps [CI = 0.4154; HI = 0.5846; RI = 0.8273; RCI = 0.3437] were retained. These trees are summarized as a SCT (S5a Fig) and a 50% MCT (S5b Fig). This analysis resulted in weak resolution in the areas among placoderms in the SCT. Interestingly, petalichthyids are closely related to ptyctodontids in the 50% MCT, back to the original results in Long et al. [20].

### MP Analysis of the Expanded Dataset

**Fig 3:**
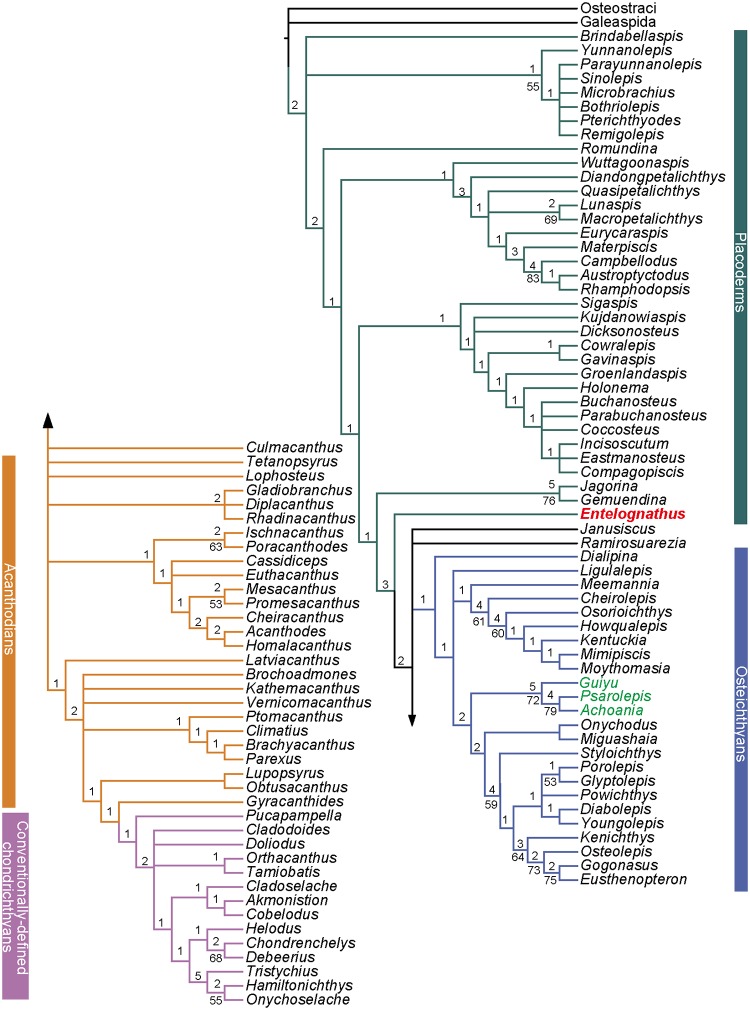
The strict consensus tree of 2496 most parsimonious trees based on the extended dataset, combining Long et al.’s dataset [20] and other recent datasets [11, 22, 33]. Numbers on branches denote Bremer and bootstrap support.

The MP analysis using the expanded dataset produces 2496 most MPTs of 955 steps (CI = 0.3770; HI = 0.6230; RI = 0.8061; RCI = 0.3039). These trees are summarized as an SCT (Fig 3) and a 50% MCT (S6 Fig). One MPT, which agrees well with the 50% MCT, is selected for illustrating inferred character transformations at various nodes (S7 Fig).

The SCT places *Brindabellaspis* and antiarchs in a polytomy with other gnathostomes. Petalichthyids and ptyctodontids form a clade which also includes *Wuttagoonaspis* as the sister taxon to all other members in this clade. Arthrodires, which were placed as the closest relatives of crown gnathostomes in previous analysis, are sister to the clade uniting the paired taxa of *Jagorina* and *Gemuendina* and crown ganthostomes, consistent with the phylogeny of Giles et al. [22].

*Entelognathus* is recovered as the sister taxon of all crown gnathostomes, as it appeared on the phylogeny recovered from the revised dataset and as it was first described [18]. The relationships of the crown gnathostomes are not well resolved in the SCT, although the osteichthyans clade remain intact. *Janusiscus* is in a polytomy with *Lophosteus*, osteichthyans and total-group chondrichthyans, different from the result from Giles et al. [22]. The *Guiyu* lineage is placed in a polytomy with the clade uniting other sarcopterygians and the actinopterygian clade. Most acanthodians collapse into polytomies, while conventionally-defined chondrichthyans remain monophyletic. On the 50% MCT, the *Guiyu* lineage is resolved as stem sarcopterygians [18] rather than stem actinopterygians [20, 29]. *Ramirosuarezia* is placed in a monophyletic group including the acanthodian taxa *Tetanopayrus*, *Diplacanthus*, *Gladiobranchus and Rhadinacanthus* in the 50% MCT. All acanthodians are placed as stem chondrichthyans (*contra* [10]), but as a paraphyletic assemblage [18, 20].

### BI Analysis of the Expanded Dataset

**Fig 4:**
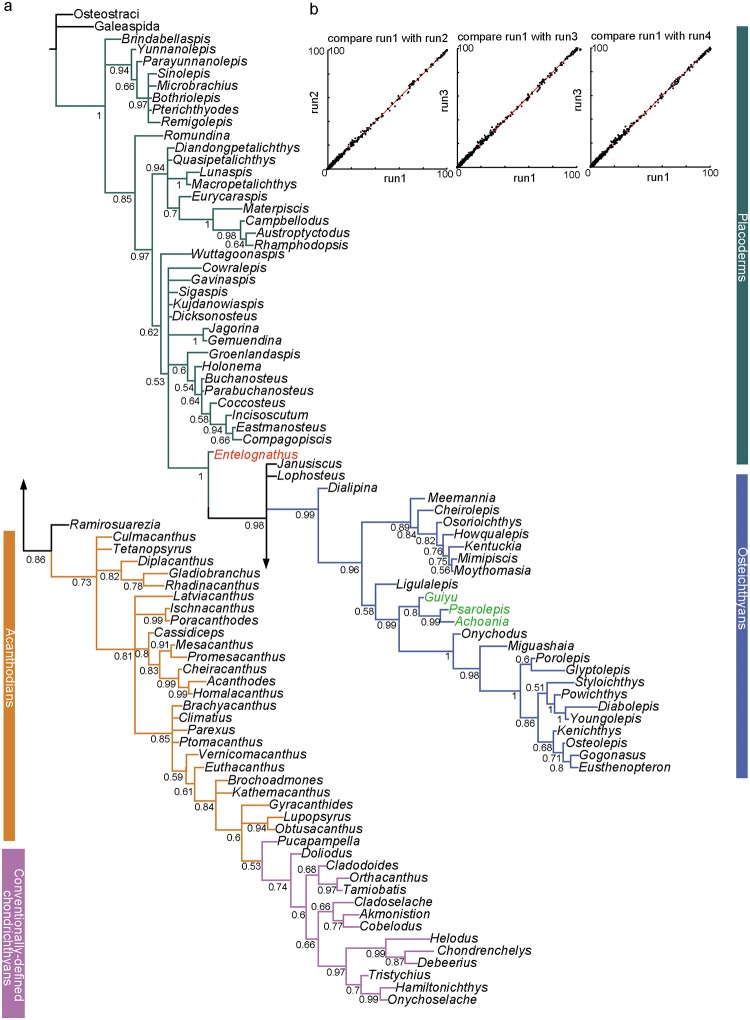
(a) Maximum clade credibility tree from the Bayesian analysis of the extended dataset, combining Long et al.’s dataset [20] and other recent datasets [11, 22, 33]. Values associated with nodes indicate the frequency with which those bipartitions occur among sampled trees. (b) Frequencies of splits found in Bayesian run 1, plotted against frequencies found in the other three runs, using AWTY [44]; all splits occurred at similar frequencies across all runs.

The BI analysis (Fig 4a) places antiarchs as the sister group to all other gnathostomes. *Brindabellaspis* and *Romundina*, together with the sister pair of petalichthyids and ptyctodontids, form a series of plesions that are successive sister groups of crown gnathostome node. *Wuttagoonaspis* is assigned a position sister to a clade comprising arthrodires *Jagorina* and *Gemuendina*, and crown gnathostome. This clade was unresolved as a polytomy.

*Entelognathus* is close to the crown gnathostome node. *Janusiscus* is situated in a polytomy with *Lophosteus*, osteichthyans and total-group chondrichthyans. The *Guiyu* lineage is resolved as stem sarcopterygians.

*Ramirosuarezia* is placed as the sister taxon to all other members along the acanthodian-chondrichthyan branch. *Ramirosuarezia* has previously been compared with rhenanid placoderms and holocephalan chondrichthyans [38] and was recovered in a polytomy with *Janusiscus* and the gnathostome crown [22]. All acanthodians fall into the stem segments of the chondrichthyan total group.

### ML Analysis of the Expanded Dataset

**Fig 5:**
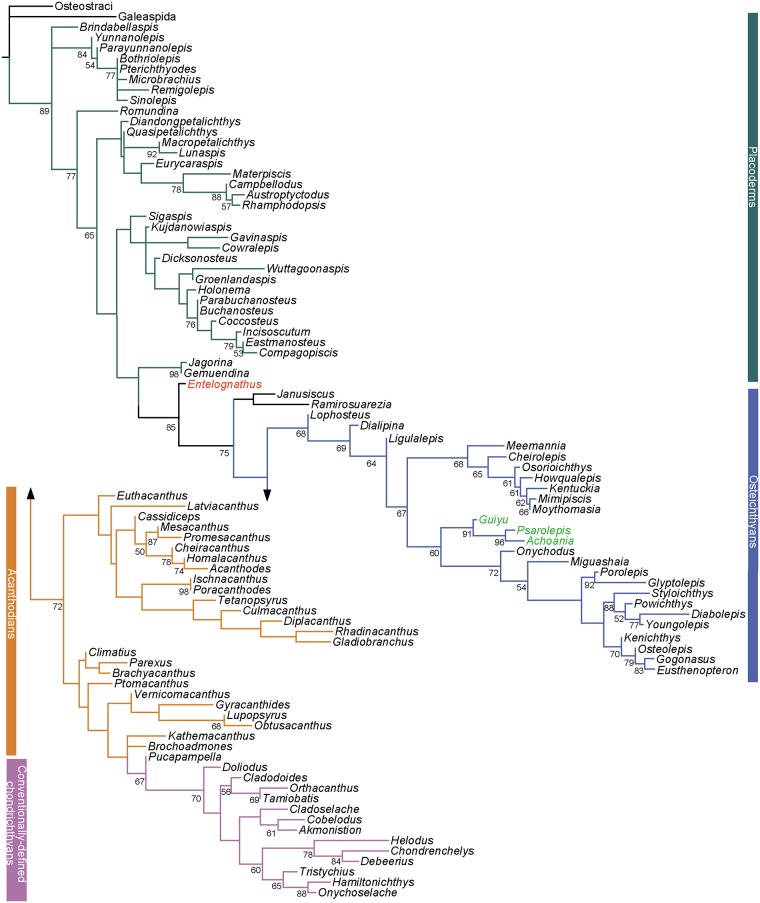
Maximum likelihood analysis of the extended dataset, combining Long et al.’s dataset [20] and other recent datasets [11, 22, 33]. Values associated with nodes indicate the likelihood bootstrap value.

The ML analysis (Fig 5) places *Brindabellaspis* and antiarchs in a polytomy with the main lineage including other gnathostomes. *Romundina* is the sister taxon to all other members of this main lineage. The sister pair of petalichthyids and ptyctodontids is retained. *Wuttagoonaspis* is assigned to the arthrodire cluster, as the sister taxon of *Groenlandaspis* and closely related to the brachythoracids.

*Entelognathus* is close to the crown gnathostome branch, as in the BI analysis tree. The clade comprising *Janusiscus* and *Ramirosuarezia* is sister to the crown gnathostomes. The *Guiyu*-lineage is resolved as stem sarcopterygians.

*Lophosteus* is placed as the sister taxon of all other members of total-group osteichthyans. All acanthodians collapse and fall into the stem segments of the chondrichthyan total group.

### Consensus of the Three Different Methods (MP, BI and ML)

Based on the expanded dataset, the three phylogenetic methods come to a consensus on the following solutions.

*Entelognathus* is placed as the sister group of crown gnathostomes. The clade uniting *Entelognathus* and crown gnathostomes is supported by the Bremer index of 3; this clade is supported by a posterior probability of 1 in the Bayesian tree and bootstrap value of 0.88 in the RAxML analysis. The crown gnathostome clade, excluding *Entelognathus*, is supported by a Bremer index of 2, a posterior probability of 0.98 in the Bayesian tree and bootstrap value of 0.75 in the RAxML analysis. The clade uniting *Entelognathus* and crown gnathostomes in one of the MPTs is supported by four homoplastic characters and one unique character (Character 244: metapterygoid portion of palatoquadrate with developed medial ventral protrusion). The reinstatement of *Entelognathus* as a stem gnathostome supports the hypothesis that marginal jaw bones (premaxilla, maxilla and dentary) and operculogular series are possibly present in the common ancestor of chondrichthyans and osteichthyans, and have been secondarily lost in chondrichthyans.

The *Guiyu* lineage is placed in the sarcopterygians rather than the actinopterygians. All the MPTs support the placement of *Guiyu* lineage as stem sarcopterygians. The clade comprising the *Guiyu* lineage and other sarcopterygians is supported by five homoplastic characters and one unique character (Character 221: unconstricted cranial notochord). The placement *Guiyu* lineage on the sarcopterygian stem is strongly supported by a posterior probability of 0.99 in the BI analysis and a bootstrap value of 0.60 in the ML analysis.

All acanthodians are a paraphyletic assemblage of stem chondrichthyans in every analysis [18, 20] (*contra* [10]). Dupret et al. [21] placed acanthodians as the monophyletic sister group of chondrichthyans. However, as discussed by Long et al. ([20]: extended data figure 7), the reported tree in Dupret et al. [21] was not the shortest possible for their data dataset. A stem chondrichthyan status of acanthodians would imply that the common ancestor of chondrichthyans and osteichthyans carried a macromeric dermal skeleton, that the macromeric skeleton is homologous in placoderms and osteichthyans, and that it has been replaced by a micromeric skeleton in chondrichthyans [18].

The relationships of petalichthyids and ptyctodontids remain uncertain. This group is supported by two unambiguous characters herein: sensory canals enclosed within dermal bones (Character 15) and no pineal perforation in skull roof (Character 24). The BI analysis results in relatively low support for this group (0.6 posterior probability) and the ML analysis returns a bootstrap value of 0.57. This pairing was found by Long et al. (2015) with 85 taxa included and the 50% MCT resulted from the revised total dataset (91 taxa included) (S5b Fig). Zhu et al. [18] assigned ptyctodontids crownward to the petalichthyid *Macropetalichthys*. Our analyses suggest that the close relationship of petalichthyids and ptyctodontids remains plausible, although the sensitivity of this pairing to minor changes in the dataset or analysis suggests that the evidence is weak at best.

### Incongruence among Different Methods

The main controversy of early gnathostome phylogeny using different methods mainly relates to the positions of the placoderm *Wuttagoonaspis*, the stem chondrichthyan *Ramirosuarezia*, and the stem osteichthyan *Lophosteus*–taxa that are either poorly known or highly mosaic in character complement. The nodes around these taxa are either poorly resolved or weakly supported within each method.

*Wuttagoonaspis* is resolved as the sister taxon of the clade uniting petalichthyids and ptyctodontids in the MP method. The clade is supported by two unambiguous homoplastic characters: central dermal skull bone (nuchal) with converging but not meeting posterior pit-line canals and supraorbital canals (Character 248); cheek plate divided (Character 278). It is placed as the sister taxon to the clade uniting arthrodires, *Jagorina*, *Gemuendina* and other crown gnathostomes in the BI tree. *Wuttagoonaspis* is assigned in the arthrodire cluster, as the sister taxon of *Groenlandaspis* and closely related to the brachythoracids in the ML tree. *Wuttagoonaspis* is an unusual form from Australia [74, 75, 122], which has been compared to petalichthyids [74]. However, these similarities have been disputed [30] and it is generally agreed that *Wuttagoonaspis* shares specialized features with arthrodires [74, 122–124] but could not be accomodated within a certain arthrodire classification. It is placed as the sister group of phyllolepids ([123]: three of six the MPTs, figure 1; [125]), or grouped with arctolepids ([123]: three of six the MPTs, figure 1). Long et al. [20] placed *Wuttagoonaspis* within the brachythoracid clade. By comparison, *Wuttagoonaspis* is resolved as the sister taxon of all other members in a major clade grouping most gnathostomes excluding *Brinabellaspis* and antiarchs in the revised results (Fig 2a). The unstable placement of *Wuttagoonaspis* is most likely caused by conflicting character sets suggesting different relationships, caused by convergent evolution with other specialised groups. Deletion of this taxon has no impact on the tree topology under the MP method.

**Fig 6:**
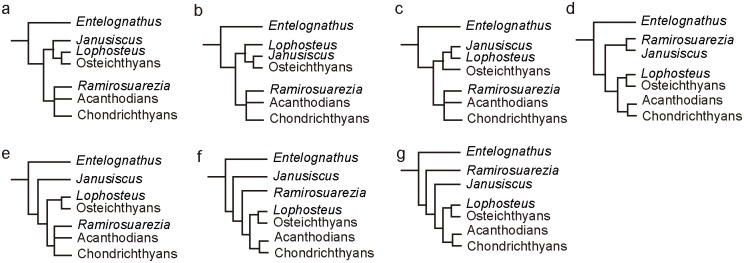
Seven different simplified phylogeny hypotheses at the crown gnathostome node based on the 2496 MPTs recovered from the extended dataset.

*Ramirosuarezia* is placed in a monophyletic group including acanthodian taxa *Tetanopsyrus*, *Diplacanthus*, *Gladiobranchus* and *Rhadinacanthus* in 92% of the MPTs (S6 Fig). The other 8% MPTs recover *Ramirosuarezia* as a stem gnathostome either as the successive plesion to or the sister group of *Janusiscus* (Fig 6), the latter result also found in Giles et al. [22]. The BI analysis (Fig 4) places *Ramirosuarezia* as the sister taxon to all other membersin the acanthodian-chondrichthyan branch. The ML analysis (Fig 5) recovers *Ramirosuarezia* and *Janusiscus* in a clade, as the sister of crown gnathostomes. *Ramirosuarezia* is an enigmatic gnathostome with characteristics in common with elasmobranch and holocephalan chondrichthyans, some placoderms and osteichthyans [54]. It was stated that no character clearly supports affinities of *Ramirosuarezia* to any of the currently known major gnathostome groups [54] and therefore its phylogenetic instability is hardly a surprise. There cannot be said to be much evidence supporting a relationship of *Ramirosuarezia* to particular acanthodians, as found in the MP analysis, as the character sets that can be scored for these two groups are largely non-overlapping. Placement of *Ramirosuarezia* within acanthodians is largely due to a single character (presence of pronounced dorsal process on Meckelian bone or cartilage), shared with *Diplacanthus*, *Tetanopsyrus* and *Gladiobranchus*. The deletion of *Ramirosuarezia*, which has more than 85% missing data, does not affect the tree topology using the MP method. Thus the unstable placement of *Ramirosuarezia* might be caused by its incompleteness and the difficulty of interpreting its characters against any model.

*Lophosteus* is situated in a polytomy with *Janusiscus*, osteichthyans and total- group chondrichthyans in the MP and BI trees. The ML analysis recovers *Lophosteus* as a member of total- group osteichthyans (Fig 5), with a bootstrap value of 68. *Lophosteus* is known from very limited materials including a fragment of jaw bone, some isolated scales, teeth and minute bone fragments [86–91]. Because of the limited knowledge about this genus and its mosaic character combination, the affinity of *Lophosteus* has long been debated. It was referred to osteichthyans on the basis of its diamond-shaped scales when it was first discovered [126, 127]. On the basis of histological information, Burrow [89] later reported its possible affinities with placoderms. Its large fin spines also suggested a relationship to acanthodians [87, 88, 90]. When the denticle-bearing jaw bone was described, it was noted that *Lophosteus* may be more closely related to crown-group osteichthyans [91]. The unresolved position of *Lophosteus* in the MP and BI trees might be due to its large proportion of missing data (93.7%). Although its position on the strict consensus tree is unresolved, all the 2496 MPTs confirm its affinities to stem osteichthyans (Fig 6). If *Lophosteus* is removed from the expanded dataset, the resolution among the stem osteichthyans becomes better—*Dialipina*, and *Ligulalepis* form two plesions that are successive sister groups of crown osteichthyans.

### Multiple Phylogenetic Methods for Paleontological Data

The MP method is generally used in phylogenetic analyses of paleontological morphological data. While the BI and ML methods are widely used to analyze molecular data, they are comparatively less explored in exclusively morphological data, paleontological data in particular. This is partly due to the small size of most palaeontological dasets which reduces the robustness of parameter estimates. Recently, the sizes of some paleontological datasets are growing (e.g. [128]), due to the accumulation of fossil materials and technique advances (e.g. high resolution computed tomography). On the other hand, some researchers have followed the ‘total evidence’ approach [129], and treated all available characters simultaneously in a single dataset or combined morphology-only datasets with DNA datasets (e.g. [129–131]). Recently, there has been an increase in the application of these probability-based approaches to paleontological morphological data.

The BI and ML methods adopt evolutionary models to evaluate the probability of character change along branches in order to determine the tree topology [34–36]. However, while the ML method determines the single tree that maximizes the probability of the observed data, and bootstrap values are applied to evaluate the robustness of the recovered clades, the BI method relies on the posterior distribution—the probability of the hypothesis given the data and a specified prior probability, posterior probability being the measure of credibility of the recovered clades. Although MP, BI and ML methods are based on different principles, the overall tree topologies recovered by three methods are often similar [132]. It is also noted that the results of an MP analysis can be reproduced by a ML approach in which each character has its own set of branch lengths [133]. Some systematists therefore argue that the usage of multiple analytical methods is largely redundant [132]. However, this is not found in the study presented here.

Firstly, the consistent tree topologies recovered by different methods suggest that the BI and ML approaches do not perform worse than the MP method. At present, the most widely used model in both BI and MP for estimating phylogeny from discrete dataset is the Mkv model (where k is the number discrete character states, and v refers to likelihood calculations being conditioned on only variable characters being present in the data) proposed by Lewis [42]. This updated model is modified from the Jukes–Cantor (JC69) model of nucleotide sequence evolution and was specifically designed to imitate the step-counting method of MP under a likelihood framework. This model assumes a Markov process for character change, allowing for multiple character-state changes along a single branch. Historically, this model has been argued that even in ideal cases in which the evolutionary model perfectly fits the assumed model of the probabilistic method, there are particular cases or biases that affect the efficiency of both ML [134] and BI [135]. Recently, the BI and ML approaches are proved to outperform parsimony for estimation of phylogeny from discrete morphological data [136], whereas, O’Reilly et al. [137] stated that Bayesian methods outperform parsimony but at the expense of topology resolution. Here, based on the tree topologies recovered by BI, ML and MP, the tree resolution of BI and ML are better than MP method. Therefore, we conduct that the BI and ML approaches could be useful in morphological phylogenetics.

Secondly, the BI and ML methods are statistically well understood and could provide biologists with not only better resolution of trees with which to formulate evolutionary hypotheses [138], but also new ways to study evolution [139]. This includes the estimation of divergence times to produce time-calibrated phylogenies [140], which can be produced using only morphological data [141], and optimization of continuous characters [142]. BI also allows the inclusion of phylogenetic uncertainty in comparative analysis [143]. Most phylogenetic comparative methods do not account for uncertainties in phylogenies and intraspecific variations. Not accounting for these sources of uncertainty leads to false perceptions of precision [144].

Thirdly, there are always conflicts among the results of the MP, ML and BI methods [34, 128]. The discrepancies might be invoked as ancillary stability measures. As to our dataset, the discrepancies around *Wuttagoonaspis*, *Ramirosuarezia* and *Lophosteus* require more thorough morphological investigation of these relevant taxa. The BI and ML methods have been developed as alternatives to parsimony reconstruction, and could reveal a surprising amount of uncertainty in ancestral reconstructions [145]. Furthermore, although these methods usually achieve concordant topological results, they may generate discordant inferences of character evolution from the same dataset [146]. This indicates that method selection has profound impacts in evolutionary scenarios and taxonomic arrangements. Given that different performances of each phylogenetic approach, our study provides an empirical case that the multiple phylogenetic analyses of morphological data are mutually complementary rather than redundant.

After all, an accurate data dataset is the foundation of any phylogenetic analysis. It is important to first make a more thorough investigation on the morphology of relevant taxa and their associated character states before any phylogenetic analysis. A long-standing idea holds that autapomorphies should be added to the data set. Müller and Reisz [147] noted that Mk model only worked well when morphological data sets incorporated autapomorphies. However, this can be problematic because it is untestable if all potential autapomorphies for a given taxon have been documented. This is particularly troublesome with fossil data, especially when dealing with taxa that are known from fragmentary material or only known from one individual. Alternatively, Müller and Reisz [147] noted that if a gamma parameter was included to account for rate variation, then Mk worked well with or without autapomorphies.

## Conclusions

The revised data from a recent phylogenetic study [20] recover *Entelognathu*s as a stem gnathostome rather than a stem osteichthyan, and the *Guiyu* lineage as stem sarcopterygians rather than stem actinopterygians.

An expanded dataset with 103 taxa and 335 characters, which is the most comprehensive morphological dataset for early gnathostomes constructed to date, is presented.

Both the parsimony and probability-based studies based on the expanded dataset of early gnathostomes confirm the paraphyly of placoderms with respect to crown gnathostomes, and place all acanthodians as a paraphyletic stem group of the conventionally defined chondrichthyans. However, some discrepancies among different methods, including the placements of *Ramirosuarezia*, *Lophosteus*, and *Wuttagoonaspis*, indicate focus areas for further research in the study of early vertebrates. Many areas of the phylogeny are highly unstable. For example, among placoderms, a number of core groups are clearly monophyletic, incuding brachythoracid arthrodires, antiarchs and ptyctodontids. However, their relationships to each other are highly unstable, analogous to the poor ability of morphological data to resolve the relationships of the orders of mammals [148] or birds [34, 128]. Accurate resolution of the true tree of relationships of placoderms may not be possible on current evidence, but Bayesian phylogenetic methods at least allow this uncertainty to be accounted for when the tree is used to study evolutionary process.

A better knowledge of the influence of different phylogenetic analyses is surely needed. The present study shows that the combination of parsimony analysis, Bayesian and maximum likelihood inferences are mutually complementary rather than redundant.

## Supporting Information

S1 Dataset Dataset of Long et al. [20] with minor revisions. (NEX)

S2 Dataset The revised dataset of Long et al. [20]. (NEX)

S3 Dataset The revised dataset of Long et al. [20] with deletion of six taxa. (NEX)

S4 Dataset Expanded dataset combining the data matrices of Long et al. [20], Giles et al. [22], Brazeau and de Winter [11], and Lu et al. [33]. (NEX)

S1 Fig The strict consensus tree and the 50% majority consensus tree of 4964 MPTs based on the original dataset from Long et al. (2015) with minor revisions. (PDF)

S2 Fig The 50% majority consensus tree of 36 MPTs based on the dataset revised from Long et al. (2015) (85 taxa). (PDF)

S3 Fig Tracing of characters changing unambiguously on one of the MPTs using the revised original dataset. (PDF)

S4 Fig The strict consensus tree of 108 MPTs based on the original dataset from Long et al. (2015) (total 91 taxa). (PDF)

S5 Fig The strict consensus tree and the 50% majority consensus tree of 532 MPTs based on the revised dataset from Long et al.’s (2015) (total 91 taxa). (PDF)

S6 Fig The 50% majority consensus tree of 2496 MPTs based on the extended dataset. (PDF)

S7 Fig Tracing of characters changing unambiguously on one of the MPTs using the extended dataset. (PDF)

S1 Text Supplementary information (revisions, taxa sources and character and taxon list). (DOCX)

S2 Text The expanded dataset and commands used in MrBayes. (TXT)

S3 Text The RAxML commands used in the maximum likelihood analysis. (TXT)

## References

[1] Nelson JS. Fishes of the World (4th edition). New York: Wiley; 2006.

[2] Moy-Thomas JA, Miles RS. Palaeozoic fishes. London: Chapman and Hall; 1971.

[3] Greenwood PH, Miles RS, Patterson C. Interrelationships of fishes. Zool J Linn Soc. 1973;53:1–536.

[4] Brazeau MD, Friedman M. The origin and early phylogenetic history of jawed vertebrates. Nature. 2015;520(7548):490–7. 10.1038/nature14438 . 25903631 PMC4648279

[5] Maisey JG. A primitive chondrichthyan braincase from the Middle Devonian of Bolivia In: Ahlberg PE, editor. Major events in early vertebrate evolution: palaeontology, phylogeny, genetics and development. London: Taylor & Francis; 2001 pp. 263–288.

[6] Miller RF, Cloutier R, Turner S. The oldest articulated chondrichthyan from the Early Devonian peroid. Nature. 2003;425:501–504. 14523444 10.1038/nature02001

[7] Miles RS. Relationships of acanthodians In: Greenwood PH, Miles RS, Patterson C, editors. Interrelationships of Fishes. London: Academic Press; 1973 pp. 63–103.

[8] Jarvik E. The systematic position of acanthodian fishes In: Andrews SM, Miles RS, Walker AD, editors. Problems in vertebrate evolution. London: Academic Press; 1977 pp. 199–225.

[9] Brazeau MD. The braincase and jaws of a Devonian ‘acanthodian’ and modern gnathostome origins. Nature. 2009;457:305–308. 10.1038/nature07436 19148098

[10] Davis SP, Finarelli JA, Coates MI. *Acanthodes* and shark-like conditions in the last common ancestor of modern gnathostomes. Nature. 2012;486(7402):247–250. 10.1038/nature11080 22699617

[11] Brazeau MD, de Winter V. The hyoid arch and braincase anatomy of Acanthodes support chondrichthyan affinity of 'acanthodians'. Proc R Soc B. 2015;282:20152210 10.1098/rspb.2015.2210. 10.1098/rspb.2015.2210 26674952 PMC4707761

[12] Miles RS. Jaw articulation and suspension in *Acanthodes* and their significance In: Ørvig T, editor. Current problems of lower vertebrate phylogeney nobel symposium 4. Stockholm: Almqvist & Wiksell; 1968 pp. 109–127.

[13] Miles RS. Articulated acanthodian fishes from the Old Red Sandstone of England, with a review of the structure and evolution of the acanthodian shoulder-girdle. Bull Br Mus (Nat Hist), Geol. 1973;24(2):111–213.

[14] Denison RH. Acanthodii. Stuttgart: Gustav Fischer Verlag; 1979.

[15] Coates MI, Davis S. About the ears: *Acanthodes* re-examined and gnathostome origin re-analyzed. J Vert Paleo. 2010;30 (suppl):74A.

[16] Zhu M, Zhao W-J, Jia L-T, Lu J, Qiao T, Qu Q-M. The oldest articulated osteichthyan reveals mosaic gnathostome characters. Nature. 2009;458:469–474. 10.1038/nature07855 19325627

[17] Qiao T, Zhu M. Cranial morphology of the Silurian sarcopterygian Guiyu oneiros (Gnathostomata: Osteichthyes). Sci China, Earth Sci. 2010;53(12):1836–1848. 10.1007/s11430-010-4089-6

[18] Zhu M, Yu X-B, Ahlberg PE, Choo B, Lu J, Qiao T, et al A Silurian placoderm with osteichthyan-like marginal jaw bones. Nature. 2013;502(7470):188–193. 10.1038/nature12617 24067611

[19] Zhu M. Bone gain and loss: insights from genomes and fossils. Nat Sci Rev. 2014;1:490–497.

[20] Long JA, Mark-Kurik E, Johanson Z, Lee MS, Young GC, Zhu M, et al Copulation in antiarch placoderms and the origin of gnathostome internal fertilization. Nature. 2015;517:196–199. 10.1038/nature13825 . 25327249

[21] Dupret V, Sanchez S, Goujet D, Tafforeau P, Ahlberg PE. A primitive placoderm sheds light on the origin of the jawed vertebrate face. Nature. 2014;507:500–503. 10.1038/nature12980 24522530

[22] Giles S, Friedman M, Brazeau MD. Osteichthyan-like cranial conditions in an Early Devonian stem gnathostome. Nature. 2015 10.1038/nature14065 . 25581798 PMC5536226

[23] Yu X-B. A new porolepiform-like fish, *Psarolepis romeri*, gen. et sp. nov. (Sarcopterygii, Osteichthyes) from the Lower Devonian of Yunnan, China. J Vert Paleo. 1998;18(2):261–274.

[24] Zhu M, Yu X-B, Janvier P. A primitive fossil fish sheds light on the origin of bony fishes. Nature. 1999;397:607–610.

[25] Zhu M, Yu X-B, Ahlberg PE. A primitive sarcopterygian fish with an eyestalk. Nature. 2001;410:81–84. 11242045 10.1038/35065078

[26] Basden AM, Young GC, Coates MI, Ritchie A. The most primitive osteichthyan braincase? Nature. 2000;403:185–188. 10646601 10.1038/35003183

[27] Friedman M, Brazeau MD. A reappraisal of the origin and basal radiation of the Osteichthyes. J Vert Paleo. 2010;30(1):36–56.

[28] Qu Q-M, Haitina T, Zhu M, Ahlberg PE. New genomic and fossil data illuminate the origin of enamel. Nature. 2015;526(7571):108–11. 10.1038/nature15259 . 26416752

[29] Schultze H-P. Scales, enamel, cosmine, ganoine, and early osteichthyans. Comptes Rendus Palevol. 2015 10.1016/j.crpv.2015.04.001

[30] Miles RS, Young GC. Placoderm interrelationships reconsidered in the light of new ptyctodontids from Gogo, Western Australia In: Andrews SM, Miles RS, Walker AD, editors. Problems in vertebrate evolution. London: Academic Press; 1977 pp. 123–198.

[31] Goujet DF. Les poissons placodermes du Spitsberg Arthrodires Dolichothoraci de la formation de Wood Bay (Dévonien inférieur). Paris: CNRS 1984.

[32] Goujet DF, Young GC. Placoderm anatomy and phylogeny: new insights In: Arratia G, Wilson MVH, Cloutier R, editors. Recent advances in the origin and early radiation of vertebrates. München: Verlag Dr. Friedrich Pfeil; 2004 pp. 109–126.

[33] Lu J, Giles S, Friedman M, den Blaauwen JL, Zhu M. The oldest actinopterygian highlights the cryptic early history of the hyperdiverse ray-finned fishes. Curr Biol. 2016 10.1016/j.cub.2016.04.045. 27212403

[34] Lee MSY, Worthy TH. Likelihood reinstates *Archaeopteryx* as a primitive bird. Biol Lett. 2011:1–5. 10.1098/rsbl.2011.0884 PMC3297401 22031726

[35] Xu X, Pol D. *Archaeopteryx*, paravian phylogenetic analyses, and the use of probability-based methods for palaeontological datasets. J Syst Palaeontol. 2013:1–12. 10.1080/14772019.2013.764357

[36] Huelsenbeck JP, Ronquist F. Mrbayes: Bayesian inference of phylogenetic trees. Bioinformatics. 2001;17(8):754–755. 11524383 10.1093/bioinformatics/17.8.754

[37] Maddison WP, Maddison DR. Mesquite: A modular system for evolutionary analysis, version 2.5. http://mesquiteproject.org; 2008.

[38] Goloboff PA, Farrisb JS, Nixon KC. TNT, a free program for phylogenetic analysis. Cladistics. 2008;24:774–786.

[39] Nixon K. WinClada ver. 1.00. 08. Published by the author, Ithaca, NY. 2002.

[40] Huelsenbeck JP, Ronquist F, Nielsen R, Bollback JP. Bayesian inference of phylogeny and its impact on evolutionary biology. Science. 2001;294:2310–2314. 11743192 10.1126/science.1065889

[41] Ronquist F, Huelsenbeck J, Teslenko M. Draft MrBayes version 3.2 manual: tutorials and model summaries. 2011:172.

[42] Lewis PO. a likelihood approach to estimating phylogeny from discrete morphological character data. Syst Biol. 2001;50:913–925. 12116640 10.1080/106351501753462876

[43] Rambaut A, Drummond AJ. TRACER v.1.5. http://beast.bio.ed.ac.uk/Tracer2004.

[44] Nylander JA, Wilgenbusch JC, Warren DL, Swofford DL. AWTY (are we there yet?): a system for graphical exploration of MCMC convergence in Bayesian phylogenetics. Bioinformatics. 2008;24(4):581–583. 17766271 10.1093/bioinformatics/btm388

[45] Stamatakis A. RAxML Version 8: A tool for phylogenetic analysis and post-analysis of large phylogenies. Bioinformatics. 2014;30(9):1312–1313. 10.1093/bioinformatics/btu033 24451623 PMC3998144

[46] Zhang M-M. Preliminary note on a Lower Devonian antiarch from Yunnan, China. Vert PalAsiat. 1980;18(3):179–190.

[47] Zhu M. The phylogeny of the Antiarcha (Placodermi, Pisces), with the description of Early Devonian antiarchs from Qujing, Yunnan, China. Bull Mus Natn Hist Nat. 1996;18:233–347.

[48] Giles S, Rücklin M, Donoghue PCJ. Histology of “placoderm” dermal skeletons: Implications for the nature of the ancestral gnathostome. J Morph. 2013;274(6):627–644. 10.1002/jmor.20119 23378262 PMC5176033

[49] Liu Y-H. On a new petalichthyid, *Eurycaraspis incilis* gen. et sp. nov., from the Middle Devonian of Zhanyi, Yunnan In: Chang M-M, Liu Y-H, Zhang G-R, editors. Early vertebrates and related problems of evolutionary biology. Beijing: Science Press; 1991 pp. 139–177.

[50] Stensiö E. Elasmobranchiomorphi Placodermata Arthrodires In: Piveteau J, editor. Traité de Paléontologie. 4(2). Paris: Masson; 1969 pp. 71–692.

[51] Dupret V. Revision of the genus *Kujdanowiaspis* Stensiö, 1942 (Placodermi, Arthrodira, “Actinolepida”) from the Lower Devonian of Podolia (Ukraine). Geodiversitas. 2010;32(1):5–63. 10.5252/g2010n1a1

[52] Gross W. *Gemuendina stuertzi* Traquair. Neuuntersuchung. Notiz Hess Land Boden. 1963;91:36–73.

[53] Young GC. The relationships of placoderm fishes. Zool J Linn Soc. 1986;88:1–57.

[54] Pradel A, Maisey JG, Tafforeau P, Janvier P. An enigmatic gnathostome vertebrate skull from the Middle Devonian of Bolivia. Acta Zool. 2009;90:123–133. 10.1111/j.1463-6395.2008.00350.x

[55] Warren A, Currie BP, Burrow C, Turner S. A redescription and reinterpretation of *Gyracanthides murrayi* Woodward 1906 (Acanthodii, Gyracanthidae) from the Lower Carboniferous of the Mansfield Basin, Victoria, Australia. J Vert Paleo. 2000;20(2):225–242.

[56] Turner S, Burrow CJ, Warren A. *Gyracanthides hawkinsi* sp. Nov. (Acanthodii, Gyracanthidae) from the Lower Carboniferous of Queensland, Australia, with a review of gyracanthid Taxa. Palaeontology. 2005;48(5):963–1006.

[57] Schultze H-P, Zidek J. Ein primitiver Acanthodier (Pisces) aus dem Unterdevon Lettlands. Paläontol Zeitsch. 1982;56(1/2):95–105.

[58] Moy-Thomas JA. On the structure and affinities of the Carboniferous Cochliodont *Helodus simplex*. Geol Mag. 1936;73:488–503.

[59] Rayner DH. On the cranial structure of an early palaeoniscid, *Kentuckia* gen. nov. Trans R Soc Edinb Earth Sci. 1951;62(1):53–83.

[60] Andrews SM, Westoll TS. The postcranial skeleton of rhipidistian fishes excluding *Eusthenopteron*. Trans R Soc Edinb Earth Sci. 1970;68(12):391–486.

[61] Jarvik E. Middle and Upper Devonian Porolepiformes from East Greenland with special reference to *Glyptolepis groenlandica* n. sp., and a discussion on the structure of the head in the Porolepiformes. Medd om Grøn. 1972;187(2):1–307.

[62] Ahlberg PE. Paired fin skeletons and relationships of the fossil group Porolepiformes (Osteichthyes: Sarcopterygii). Zool J Linn Soc. 1989;96:119–166.

[63] Cloutier R, Ahlberg PE. Morphology, characters, and the interrelationships of basal sarcopterygians In: Stiasnny MLJ, Parenti LR, Johnson GD, editors. Interrelationships of fishes. San Diego: Academic Press; 1996 pp. 445–479.

[64] Liu T-S, P'an K. Devonian fishes from Wutung series near Nanking, China. Palaeontologica Sin. 1958;141(15):1–41.

[65] Ritchie A, Shitao W, Young GC, Guorui Z. The Sinolepidae, a family of antiarchs (placoderm fishes) from the Devonian of South China and eastern Australia. Rec Aust Mus. 1992;44(3):319–370. 10.3853/j.0067-1975.44.1992.38

[66] Hemmings SK. The Old Red Sandstone antiarchs of Scotland: *Pterichthyodes* and *Microbrachius*. Monogr Palaeontogr Soc. 1978;131:1–64.

[67] Denison RH. Placodermi. Handbook of paleoichthyology, vol 2 1978:128.

[68] Pan J, Wang S, Liu S, Gu Q, Jia H. Discovery of Devonian *Bothriolepis* and *Remigolepis* in Ningxia. Acta Geol Sin. 1980;3:176–184.

[69] Pan J, Huo F-C, Cao J-X, Gu Q-C, Liu S-Y, Wang J-Q, et al Continental Devonian system of Ningxia and its biotas. Beijing: Geological Publishing House; 1987.

[70] Johanson Z. New *Remigolepis* (Placodermi; Antiarchi) from Canowindra, New South Wales, Australia. Geol Mag. 1997;134(6):813–846.

[71] P'an K, Wang S-T. Devonian Agnatha and pisces of South China In: Symposium on the Devonian system of South China. Beijing: Geological Press; 1978 pp. 298–333.

[72] Zhu M. New information on *Diandongpetalichthys* (Placodermi: Petalichthyida) In: Chang M-M, Liu Y-H, Zhang G-R, editors. Early vertebrates and related problems of evolutionary biology. Beijing: Science Press 1991 pp. 179–194.

[73] Liu Y-H. On the new forms of Polybranchiaspiformes and Petalichthyida from Devonian of South—West China. Vert PalAsiat. 1973;11(2):132–143.

[74] Ritchie A. *Wuttagoonaspis* gen. nov., an unusual arthrodire from the Devonian of Western New South Wales, Australia. Palaeontogr Abt A. 1973;143:58–72.

[75] Young GC, Goujet D. Devonian fish remains from the Dulcie Sandstone and Cravens Peak Beds, Georgina Basin, central Australia. Rec West Aust Mus (suppl). 2003;65:1–85.

[76] Ritchie A. Groenlandaspis in Antarctica, Australia and Europe. Nature. 1975;254:569–573.

[77] Anderson ME, Hiller N, Gess RW. The first *Bothriolepis*-associated Devonian fish fauna from Africa. S Afr J Sci. 1994;90:397–403.

[78] Goujet DF. *Sigaspis*, un nouvel arthrodire du Dévonien inférieur du Spitsberg. Palaeontogr Abt A. 1973;143:73–88.

[79] Goujet DF. *Dicksonosteus*, un nouvel arthrodire du Dévonien du Spitsberg remarques sur le squelette visceral des Dolichothoraci In: Lehman JP, editor. Problèmes actuels de paléontologie-evolution des vertébrés. 218 Paris: Colloques Internationaux du CNRS; 1975 pp. 81–99.

[80] White EI. The larger arthrodiran fishes from the area of Burrinjuck Dam, N.S.W. Trans Zool Soc Lond. 1978;34:149–262.

[81] Miles RS. New Specimens of *Holonema* from the Upper Devonian of The Holonematidae (Placoderm Fishes), A review based on western Australia. Phil Trans R Soc Lond B. 1971;263:101–234.

[82] Trinajstic K, Boisvert C, Long J, Maksimenko A, Johanson Z. Pelvic and reproductive structures in placoderms (stem gnathostomes). Biol Rev. 2015 10.1111/brv.12118 . 24889865

[83] Gardiner BG, Miles RS. Eubrachythoracid arthrodires from Gogo, Western Australia. Zool J Linn Soc. 1994;112:443–477.

[84] Long JA, Trinajstic K, Young GC, Senden T. Live birth in the Devonian period. Nature. 2008;453(7195):650–652. 10.1038/nature06966 18509443

[85] Traquair RH. Notes of the nomenclature of the fishes of the Old Red Sandstone of Great Britain. Geol Mag. 1888;5:507–517.

[86] Gross W. *Lophosteus superbus* Pander, ein Teleostome aus dem Silur Oesels. Lethaia. 1969;2:15–47.

[87] Gross W. *Lophosteus superbus* Pander: Zähne, Zahnknochen und besondere Schuppenformen. Lethaia. 1971;4:131–152.

[88] Otto M. Zur systematischen Stellung der Lophosteiden (Obersilur, Pisces inc. sedis). Paläontol Zeitsch. 1991;65:345–350.

[89] Burrow CJ. A new Lophosteiform (Osteichthyes) from the Lower Devonian of Australia. Geobios M S. 1995;19:327–333.

[90] Schultze H-P, Märss T. Revisiting *Lophosteus* Pander 1856, a primitive osteichthyan. Acta Univ Latv. 2004;679:57–78.

[91] Botella H, Blom H, Dorka M, Ahlberg PE, Janvier P. Jaws and teeth of the earliest bony fishes. Nature. 2007;448(7153):583–586. 10.1038/nature05989 17671501

[92] Taverne L. *Osorioichthys marginis*, "Paléonisciforme" du Famennien de Belgique et la phylogénie des Actinoptérygiens dévoniens (Pisces). Bull Instit Roy Sci Nat Belgique, Sci Terre. 1997;67:57–78.

[93] Zhu M, Yu X-B, Wang W, Zhao W-J, Jia L-T. A primitive fish provides key characters bearing on deep osteichthyan phylogeny. Nature. 2006;441:77–80. 16672968 10.1038/nature04563

[94] Zhu M, Wang W, Yu X-B. *Meemannia eos*, a basal sarcopterygian fish from the Lower Devonian of China–expanded description and significance In: Elliott DK, Maisey JG, Yu X-B, Miao D-S, editors. Morphology, phylogeny and paleobiogeography of fossil fishes. München: Verlag Dr. Friedrich Pfeil; 2010 pp. 199–214.

[95] Zhu M, Yu X-B. Lower jaw character transitions among major sarcopterygian groups—a survey based on new materials from Yunnan, China In: Arratia G, Wilson MVH, Cloutier R, editors. Recent advances in the origin and early radiation of vertebrates. München: Verlag Dr. Friedrich Pfeil; 2004 pp. 271–286.

[96] Zhu M, Yu X-B. Stem sarcopterygians have primitive polybasal fin articulation. Biol Lett. 2009;5(3):372–375. 10.1098/rsbl.2008.0784 19324642 PMC2679918

[97] Schultze H-P. Crossopterygier mit heterozerker Schwanzflosse aus dem Oberdevon Kanadas, nebst einer Beschreibung von Onychodontida-Resten aus dem Mitteldevon Spaniens und aus dem Karbon der USA. Palaeontogr Abt A. 1973;143:188–208.

[98] Cloutier R. The primitive actinistian *Miguashaia bureaui* Schultze (Sarcopterygii) In: Schultze H-P, Cloutier R, editors. Devonian fishes and plants of miguasha, Quebec, Canada. München: Verlag Dr. Freidrich Pfeil; 1996 pp. 227–247.

[99] Forey PL. History of the coelacanth fishes. London: Chapman&Hall; 1998.

[100] Forey PL, Ahlberg PE, Luksevics E, Zupins I. A new coelacanth from the Middle Devonian of Latvia. J Vert Paleo. 2000;20(2):243–252.

[101] Zhu M, Yu X-B. A primitive fish close to the common ancestor of tetrapods and lungfish. Nature. 2002;418(6899):767–770. 12181564 10.1038/nature00871

[102] Chang M-M, Yu X-B. A new crossopterygian, *Youngolepis praecursor*, gen. et sp. nov., from Lower Devonian of E. Yunnan, China. Sci Sin. 1981;24(1):89–97.

[103] Chang M-M. The braincase of *Youngolepis*, a Lower Devonian crossopterygian from Yunnan, south-western China. Stockholm: University of Stockholm, Department of Geology; 1982.

[104] Chang M-M. Head exoskeleton and shoulder girdle of *Youngolepis* In: Chang M-M, Liu Y-H, Zhang G-R, editors. Early vertebrates and related problems of evolutionary biology. Beijing: Science Press; 1991 pp. 355–378.

[105] Chang M-M, Smith MM. Is *Youngolepis* a porolepiform? J Vert Paleo. 1992;12(3):294–312.

[106] Chang M-M. Synapomorphies and scenarios—more characters of *Youngolepis* betraying its affinity to the Dipnoi In: Arratia G, Wilson MVH, Cloutier R, editors. Recent advances in the origin and early radiation of vertebrates. München: Verlag Dr. Friedrich Pfeil; 2004 pp. 665–686.

[107] Jessen HL. A new choanate fish, *Powichthys thorsteinssoni* n.g., n.sp., from the early Lower Devonian of the Canadian Arctic Archipelago In: Lehman JP, editor. Problèmes actuels de paléontologie-evolution des vertébrés. 218 Paris: Colloques Internationaux du CNRS; 1975 pp. 213–222.

[108] Jessen HL. Lower Devonian Porolepiformes from the Canadian Arctic with special reference to *Powichthys thorsteinssoni* Jessen Palaeontogr Abt A. 1980;167:180–214.

[109] Clément G, Janvier P. *Powichthys* spitsbergensis sp. nov., a new member of the Dipnomorpha (Sarcopterygii, lobe-finned fishes) from the Lower Devonian of Spitsbergen, with remarks on basal dipnomorph anatomy. Fossils & Strata. 2004;50:92–112.

[110] Clément G, Ahlberg PE. The endocranial anatomy of the early sarcopterygian *Powichthys* from Spitsbergen, based on CT scanning In: Elliott DK, Maisey JG, Yu X-B, Miao D-S, editors. Morphology, phylogeny and paleobiogeography of fossil fishes. München: Verlag Dr. Friedrich Pfeil; 2010 pp. 363–377.

[111] Chang M-M, Zhu M. A new osteolepidid from the Middle Devonian of Qujing, Yunnan. Mem Ass Australas Palaeontols. 1993;15:16.

[112] Zhu M, Ahlberg PE. The origin of the internal nostril of tetrapods. Nature. 2004;432(7013):94–97. 15525987 10.1038/nature02843

[113] Jarvik E. Note on the Upper Devonian vertebrate fauna of East Greenland and on the age of the ichthyostegid stegocephalians. Arkiv för Zoologi. 1948;41(13):1–8.

[114] Jarvik E. Basic structure and evolution of vertebrates, Vol. 1 London: Academic Press; 1980.

[115] Janvier P. The phylogeny of the Craniata, with particular reference to the significance of fossil "agnathans". J Vert Paleo. 1981;1(2):121–159.

[116] Janvier P. Les Céphalaspides du Spitsberg: anatomie, phylogénie et systématique des Ostéostracés siluro-dévoniens; revisions des Ostéostracés de la Formation de Wood Bay (Dévonien inférieur du Spitsberg). Paris: Cahiers de Paléontologie, CNRS; 1985.

[117] Janvier P, Arsenault M, Desbiens S. Calcified cartilage in the paired fins of the osteostracan *Escuminaspis laticeps* (Traquair 1880), from the Late Devonian of Miguasha (Québec, Canada), with a consideration of the early evolution of the pectoral fin endoskeleton in vertebrates. J Vert Paleo. 2004;24(4):773–779.

[118] Halstead LB. Internal anatomy of the polybranchiaspids (Agnatha, Galeaspida). Nature. 1979;282:833–836.

[119] Wang N-Z. Two new Silurian galeaspids (jawless craniates) from Zhejiang Province, China, with a discussion of galeaspid-gnathostome relationships In: Chang M-M, Liu Y-H, Zhang G-R, editors. Early vertebrates and related problems of evolutionary biology. Beijing: Science Press; 1991 pp. 41–66.

[120] Pan J. New galeaspids (Agnatha) from the Silurian and Devonian of China. Beijing: Geological Publishing House; 1992.

[121] Gai Z-K, Donoghue PCJ, Zhu M, Janvier P, Stampanoni M. Fossil jawless fish from China foreshadows early jawed vertebrate anatomy. Nature. 2011;476:324–327. 10.1038/nature10276 21850106

[122] Rade J. Upper Devonian fish from the Mt. Jack area, New South Wales, Australia. J Paleontol. 1964;38:929–932.

[123] Goujet DF, Young GC. Interrelationships of placoderms revisited. Geobios M S. 1995;19:89–95.

[124] Dupret V, Zhu M. The earliest phyllolepid (Placodermi, Arthrodira) from the Late Lochkovian (Early Devonian) of Yunnan (South China). Geol Mag. 2008;145(2):257–278. 10.1017/s0016756807004207

[125] Long JA. New phyllolepids from Victoria, and the relationships of the group. Proc Linn Soc NSW. 1984;107:263–308.

[126] Pander CH. Monographie der fossilen Fische des silurischen systems der russischbaltischen gouvernements. St Petersburg: Akademie der Wissenschaften; 1856.

[127] Rohon JV. Die Obersilurischen Fische von Oesel. Theil 2. Mém Acad Imp Sci St Petersbourg. 1893;41:1–124.

[128] Xu X, You H, Du K, Han F. An *Archaeopteryx*-like theropod from China and the origin of Avialae. Nature. 2011;475(7357):465–470. 10.1038/nature10288 21796204

[129] Kluge AG. A concern for evidence and a phylogenetic hypothesis of relationships among Epicrates (Boidae, Serpentes). Syst Zool. 1989;38(1):7–25.

[130] Nylander J, Ronquist F, Huelsenbeck J, Nieves-Aldrey J. Bayesian phylogenetic analysis of combined data. Syst Biol. 2004;53(1):47–67. 10.1080/10635150490264699 14965900

[131] Ni X, Gebo DL, Dagosto M, Meng J, Tafforeau P, Flynn JJ, et al The oldest known primate skeleton and early haplorhine evolution. Nature. 2013;498(7452):60–64. 10.1038/nature12200 23739424

[132] Rindal E, Brower AVZ. Do model-based phylogenetic analyses perform better than parsimony? A test with empirical data. Cladistics. 2011;27(3):331–334. 10.1111/j.1096-0031.2010.00342.x 34875779

[133] Tuffley C, Steel M. Links between maximum likelihood and maximum parsimony under a simple model of site substitution. Bull Math Biol. 1997;59(3):581–607. 9172826 10.1007/BF02459467

[134] Pol D, Siddall ME. Biases in maximum likelihood and parsimony: a simulation approach to a ten-taxon case. Cladistics. 2001;17:266–281. 10.1111/j.1096-0031.2001.tb00123.x 34911243

[135] Pickett KM, Randle CP. Strange bayes indeed: uniform topological priors imply non-uniform clade priors. Mol Phy Evol. 2005;34(1):203–211. 10.1016/j.ympev.2004.09.001. 15579393

[136] Wright AM, Hillis DM. Bayesian analysis using a simple likelihood model outperforms parsimony for estimation of phylogeny from discrete morphological data. PLoS ONE. 2014: 9(10): e109210 10.1371/journal.pone.0109210 25279853 PMC4184849

[137] O’Reilly JE, Puttick MN, Parry L, Tanner AR, Tarver JE, Fleming J, et al Bayesian methods outperform parsimony but at the expense of precision in the estimation of phylogeny from discrete morphological data. Biol Lett 2016: 12: 20160081 10.1098/rsbl.2016.0081 10.1098/rsbl.2016.0081 27095266 PMC4881353

[138] Holder M, Lewis PO. Phylogeny estimation: traditional and Bayesian approaches. Nat Rev Genet. 2003;4(4):275–284. 10.1038/nrg1044 12671658

[139] Lewis PO. Phylogenetic systematics turns over a new leaf. Trends Ecol Evo. 2001;16(1):30–37. 10.1016/s0169-5347(00)02025-5 11146142

[140] Thorne JL, Kishino H. Divergence time and evolutionary rate estimation with multilocus data. Syst Biol. 2002;51(5):689–702. 10.1080/10635150290102456 12396584

[141] Lee MSY, Cau A, Naish D, Dyke G. Morphological clocks in palaeontology, and a mid-Cretaceous origin of crown Aves. Syst Biol. 2014;63(3):442–449. 10.1093/sysbio/syt110 24449041

[142] Lee MS, Cau A, Naish D, Dyke GJ. Dinosaur evolution. Sustained miniaturization and anatomical innovation in the dinosaurian ancestors of birds. Science. 2014;345(6196):562–6. . 25082702 10.1126/science.1252243

[143] de Villemereuil P, Wells JA, Edwards RD, Blomberg SP. Bayesian models for comparative analysis integrating phylogenetic uncertainty. BMC Evo Bio. 2012;12(1):102. 10.1186/1471-2148-12-102 PMC3582467 22741602

[144] Duchêne S, Lanfear R. Phylogenetic uncertainty can bias the number of evolutionary transitions estimated from ancestral state reconstruction methods. J Exp Zool (Mol Dev Evol) 2015; 324 (6): 517–524. 10.1002/jez.b.22638 26173578

[145] Cunningham CW, Omland KE, Oakley TH. Reconstructing ancestral character states: a critical reappraisal. Trends Ecol Evol. 1998;13(9):361–6. Epub 1998/09/01. . 21238344 10.1016/s0169-5347(98)01382-2

[146] Ronquist F. Bayesian inference of character evolution. Trends Ecol Evol. 2004;19(9):475–81. 10.1016/j.tree.2004.07.002 . 16701310

[147] Müller J, Reisz R. The phylogeny of early eureptiles: comparing parsimony and Bayesian approaches in the investigation of a basal fossil clade. Syst Biol. 2006;55(3):503–511. 10.1080/10635150600755396 16861212

[148] Asher RJ, Geisler JH, Sanchez-Villagra MR. Morphology, paleontology, and placental mammal phylogeny. Syst Biol. 2008;57(2):311–317. ISI:000255690200011. 10.1080/10635150802033022 18432551

