# Supplementary material for: Early Gnathostome Phylogeny Revisited: Multiple Method Consensus
Source: PLoS One. 2016 Sep 20;11(9):e0163157. doi: 10.1371/journal.pone.0163157 (PMC5029804; doi:10.1371/journal.pone.0163157)
Supplement: S1 Fig — (PDF) [file pone.0163157.s005.pdf]

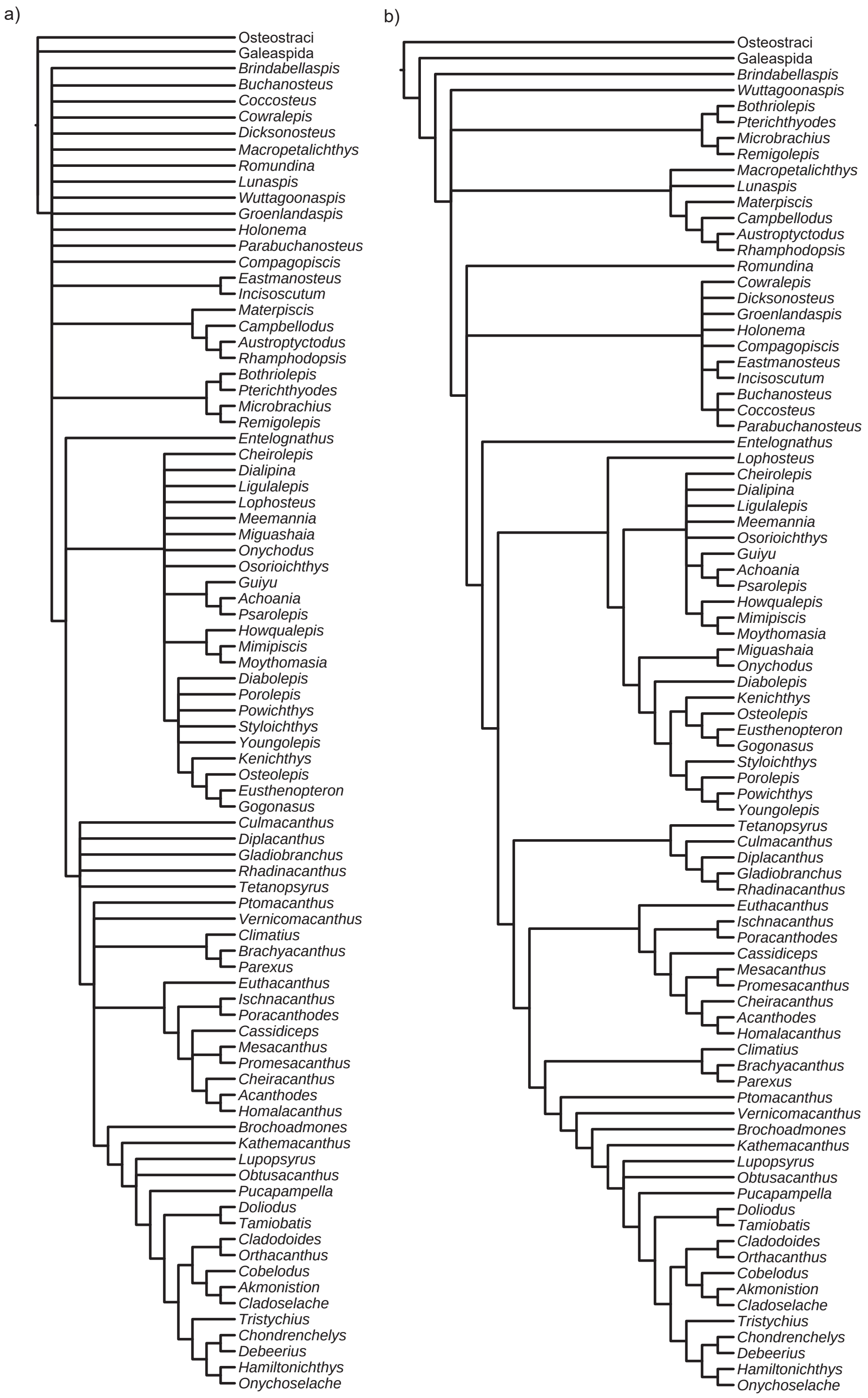

Figure S1. The strict consensus tree (a) and the 50% majority consensus tree (b) of 4964 most parsimonious trees based on the original dataset with minor revisions (6 erroneously coded characters by clerical error were revised).
