# Supplementary material for: Early Gnathostome Phylogeny Revisited: Multiple Method Consensus
Source: PLoS One. 2016 Sep 20;11(9):e0163157. doi: 10.1371/journal.pone.0163157 (PMC5029804; doi:10.1371/journal.pone.0163157)
Supplement: S2 Fig — (PDF) [file pone.0163157.s006.pdf]

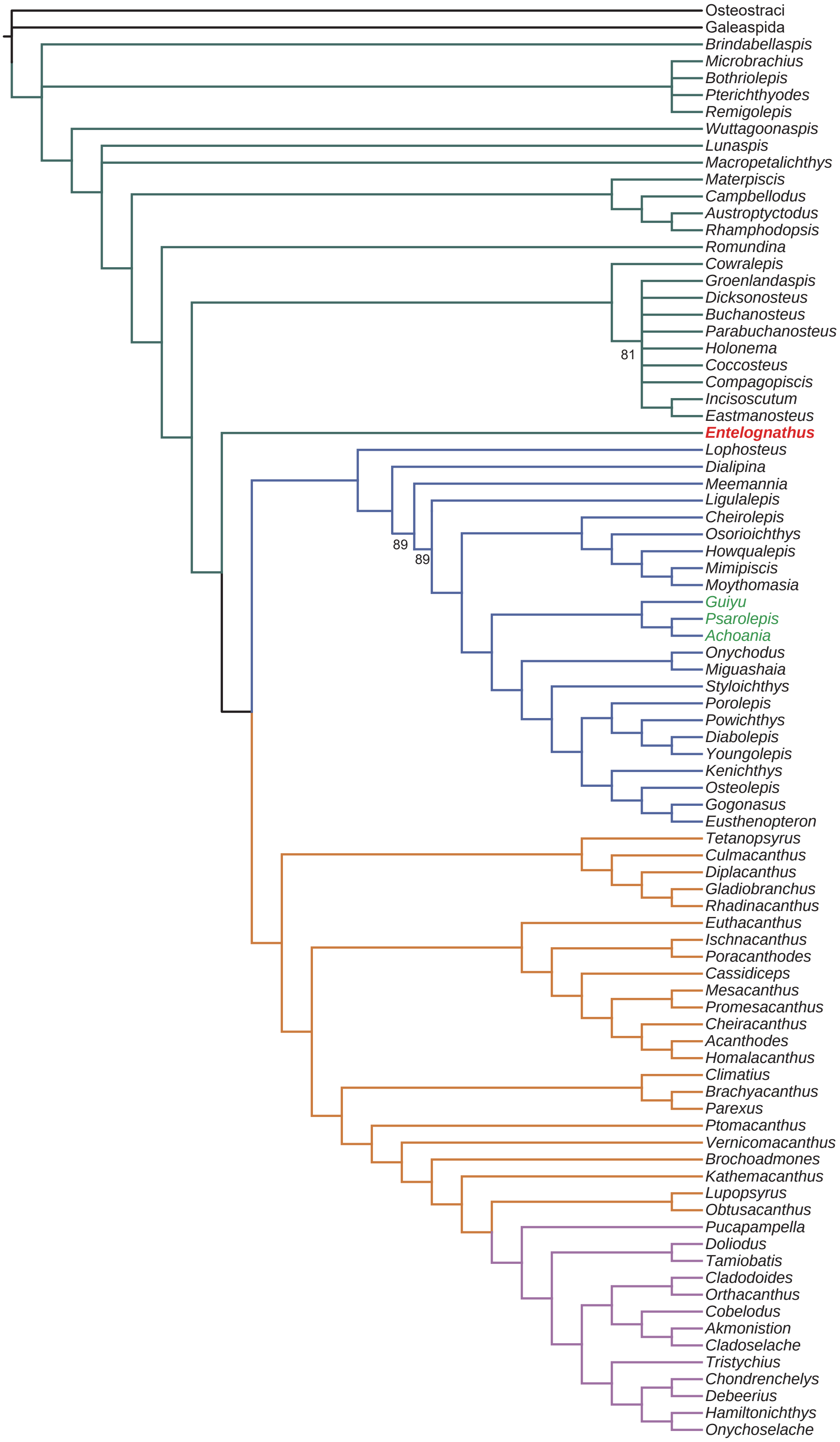

Figure S2. The 50% majority consensus tree of 36 most parsimonious trees based on the dataset revised from Long et al. (2015) (85 taxa). Numbers on branches indicate the percentage of most-parsimonious trees that contain a particular clade (100% unless otherwise indicated).
