# Supplementary material for: Early Gnathostome Phylogeny Revisited: Multiple Method Consensus
Source: PLoS One. 2016 Sep 20;11(9):e0163157. doi: 10.1371/journal.pone.0163157 (PMC5029804; doi:10.1371/journal.pone.0163157)
Supplement: S4 Fig — (PDF) [file pone.0163157.s008.pdf]

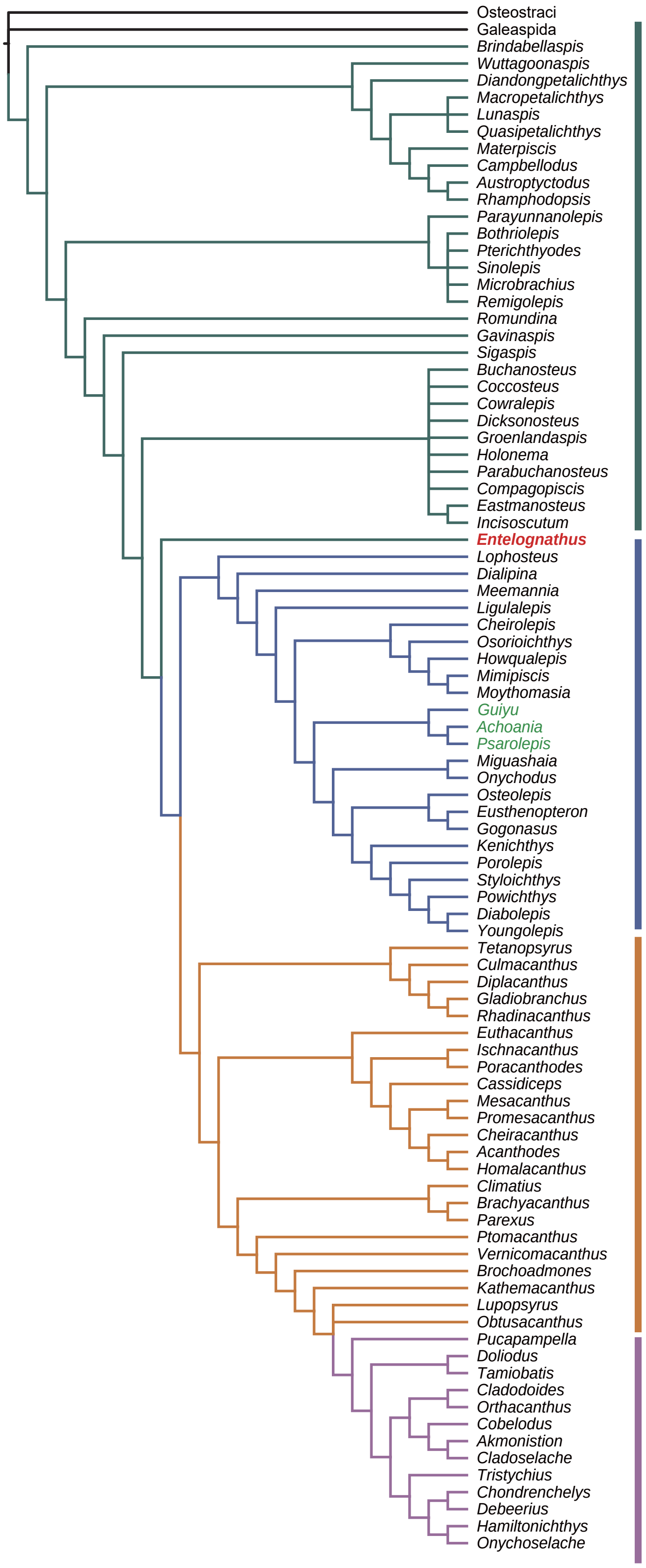

Figure S4. The strict consensus tree of 108 most parsimonious trees based on the original dataset from Long et al. (2015) (total 91 taxa).
