# Supplementary material for: Early Gnathostome Phylogeny Revisited: Multiple Method Consensus
Source: PLoS One. 2016 Sep 20;11(9):e0163157. doi: 10.1371/journal.pone.0163157 (PMC5029804; doi:10.1371/journal.pone.0163157)
Supplement: S1 Text — (DOCX) [file pone.0163157.s012.docx]

**Supplementary Information**

Part I. Revisions of Long et al. (2015)’s dataset

Part II. Taxa and principal sources of data

Part III. Character list

**Part I. Revisions of Long et al. (2015)’s dataset**

Presence or absence of perichondral bone (Character 2). Zhu et al. (2013) coded Galeaspida as “0” (presence), whereas Dupret et al. (2014) coded it as “1”. The presence of perichondral bone in galeaspids was first recorded by Zhu and Janvier (1998), and later questioned by Wang et al. (2005). We adopt Wang et al. (2005) that the superficial layer of the endoskeleton that is in contact with the dermoskeleton is an outer poorly-mineralized zone of globular calcified cartilage. However, this absence of perichondral bone along the boundary between dermoskeleton and endoskeleton cannot exclude the possibility that the free surface of endoskeleton (e.g. the ventral and lateral walls of braincase, the walls of branchial arches) is composed of perichondral bone lining. The remarkable resemblances between osteostracan and galeaspid neurocrania in preservation type favour this possibility. Moreover, in our Xiaoxiang Fauna collection, the superficial lining of endoskeleton in galeaspids has the same preservation as the perichondral bone of osteichthyans whose nature is beyond question. Considering this uncertainty, before the histological evidence in galeaspids for this free surface is provided, we code Galeaspida as “?” or polymorphic for conservation. Coding Galeaspida either as “0” or “1” will not impact the phylogenetic results.

Body scale profile (Character 10): *Brindabellaspis* (Young 1980) is unknown; *Psarolepis* with distinct crown and base demarcated by a constriction (neck) (0) (Qu et al. 2013)

Body scales with flattened base (Character 12): *Psarolepis* is coded as absent (1) (Qu, et al. 2013).

Sensory line network (Character 15): *Brindabellaspis* (Young 1980) and *Microbrachius* (Hemmings 1978) are coded as preserved as open grooves (0); *Diandongpetalichthys* is coded as passing through canals enclosed within dermal bones (1) (P'an and Wang 1978; Zhu 1991).

Nasal opening(s) (Character 55): *Holonema* is revised from 0 (dorsal, placed between orbits) to 1 (ventral and anterior to orbits) (Miles 1971a, b).

Orbit dorsal or facing dorsolaterally, surrounded laterally by endocranium (Character 65): *Diandongpetalichthys* (P'an and Wang 1978; Zhu 1991)*, Quasipetalichthys* (Liu 1973) and *Materpiscis* (Long et al. 2008) are coded as absent (1); *Lunaspis* (Heintz 1937; Gross 1961), *Microbrachius* (Hemmings 1978) and *Sinolepis* (Liu and P'an 1958) are coded as present (0).

Narrow interorbital septum (Character 67): present in *Yunnanolepis* (Zhu 1996)and *Parayunnanolepis* (Zhang et al. 2001); absent in *Entelognathus* (Zhu et al. 2013).

Short otico-occipital region of braincase (Character 71): *Sinolepis* (Liu and P'an 1958), *Remigolepis* (Johanson 1997), *Gavinaspis* (Dupret and Zhu 2008) and *Microbrachius* (Hemmings 1978) should be coded *“*0” (absent) as in other placoderms and *Gogonasus* should be “1” (present).

Basicranial morphology (Character 73) in *Entelognathus* is platybasic (0) (Zhu, et al. 2013).

Position of hyomandibula articulation on neurocranium (Character 76) is “unknown” in *Yunnanolepis* (Zhu 1996).

Dermal shoulder girdle composition (Character 101): *Pterichthyodes* (Hemmings 1978) should be ventral and dorsal (scapular) components (0).

Dermal shoulder girdle forming a complete ring around the trunk (Character 102): *Guiyu* shoule be coded as “1” (absent) (Zhu et al. 2009); *Vernicomacanthus* is unavailable (Miles 1973).

Fin base articulation on scapulocoracoid (Character 115) is stenobasal (0) in *Yunnanolepis* (Zhu 1996).

Paired fin spines (Character124): The spinal plates of placoderms were treated as homologues of pectoral fin spines of acanthodians and chondrichthyans. Accordingly, the codings in many placoderm taxa, were changed to “1” (presence)

Fin spines with ridges (Character 128): Zhu et al. (2013) had a refined formulation for this character, and restricted the character to pectoral fin spines. Following Brazeau (2009, Character 125) and Davis et al. (2012, Character 128), the spinal plates of placoderms were treated as homologues of pectoral fin spines of acanthodians and chondrichthyans. Accordingly, the codings in many placoderm taxa, *Guiyu* and *Psarolepis* were changed back to “1” (presence). Galeaspids were coded as “-” (unavailability), following Zhu et al. (2013).

Fin spines with nodes (character 129), and Fin spines with rows of large retrorse denticles (character 130): spinal plate is regarded as spines in placoderms and *Entelognathus*. Thus the codings in these taxon have been revised based on the features on their spinal plates.

Median gular (Character 135) present in *Yunnanolepis* (Zhu 1996), *Kujdanowiaspis* (Stensiö 1969; Dupret 2010), *Jagorina* (Stensiö 1969), and *Gemuendina* (Gross 1963; Young 1986) have been corrected. Unknown, absent, or logical impossibility in these taxa.

Number of marginal bones alongside paired median skull roofing bones over the otico-occipital division of braincase (Character 143) is two or more (1) in placoderms *Yunnanolepis* (Zhu 1996)*, Eurycaraspis* (Liu 1991) *and Kujdanowiaspis* (Stensiö 1969; Dupret 2010).

Type of dermal neck-joint (Character 144): *Yunnanolepis* and *Parayunnanolepis* (Zhang, et al. 2001) was coded as “2” (reversed ginglymoid).

Shape of parasphenoid denticulated field (Character 155): Zhu et al. (2013) identified three states for this character. By oversight, Dupret et al. (2014) mixed states 1 and 2 of this character as a single state, and the third state (slender, splint-shaped parasphenoid, state 2 in Zhu et al., 2013) was not coded. The formulation and state codings of this character were restored from Zhu et al. (2013).

Resorption and redeposition of odontodes (Character 157): Dupret et al. (2014) missed the codings of the second state (developed resorption and redeposition of odontodes) in their dataset, thus rendered this character uninformative. The codings of the second state were restored from Zhu et al. (2013).

Tectal (Character 166): the codings of placoderms are revised based on the comparisons between placoderms and osteichthyans (Zhu et al. 2013).

Paranuchal number (Character 171) : *Diandongpetalichthys* should be coded “1” (two pairs) (P'an and Wang 1978; Zhu 1991).

Large unpaired median bone contributing to posterior margin of skull roof (Character 172) is absent in *Howqualepis, Mimipiscis and Moythomasia.*

Contact of nuchal or centronuchal plate with paired preorbital plates (*Character 173* ) is unavailable in *Microbrachius, Sinolepis and Remigolepis.*

Dermal neck-joint between paired main-lateral-line-bearing bones of skull and shoulder girdle (Character 177): *Macropetalichthys* and *Romundina* were coded as “1” (presence).

Course of ethmoid commissure (Characters 188) and Parasymphysial plate (Character 211): Zhu et al. (2013) identified three states for each character. By oversight, Dupret et al. (2014) mixed states 0 and 1 of each character as a single state, and rendered their codings confused. The formulation and state codings of these two character were restored from Zhu et al. (2013).

Posterior end of supraorbital canal (character 194): Dupret et al. (2014) redefined this character and identified five states. The codings of placoderms are revised based on the comparisons between placoderms and osteichthyans (Zhu et al. 2013).

Contact of supraorbital and infraorbital canals (Character196): *Brindabellaspis* (Young 1980) is unknown ; supraorbital and infraorbital canals of *Romundina* are contacted rostrally (0) (Ørvig 1975)*.*

Tooth-bearing median rostral (Character203) is absent (0) in *Entelognathus* (Zhu, et al. 2013).

Opercular suspension on braincase (Character 224): opercular cartilage is absent (0) in antiarchs *Yunnanolepis* (Zhu 1996), *Parayunnanolepis* (Zhang, et al. 2001), and *Bothriolepis* (Young 1984).

Parasphenoid denticle field with anteriorly divergent lateral margins (Character229): *Bothriolepis* (Young 1984) and *Parayunnanolepis* (Zhang, et al. 2001) are coded as absent (0).

Dorsal cleithrum (AL of the Placodermi), ventral cleithrum (AVL of the Placodermi) and pectoral spine (SP of the Placodermi) (Character234) is not fused (0) in *Entelognathus* (Zhu, et al. 2013) and *Romundina (Ørvig 1975).*

Number of sclerotic plates (character 241): osteostracans have 5 sclerotic plates (ref: ) (1: more than four); *Bothriolepis* (Arsenault et al. 2004), *Parayunnanolepis* (Zhang, et al. 2001) and *Remigolepis* (Johanson 1997) bear three sclerotic plates (0: four or less).

Many characters in *Vernicomacanthus* (Miles 1973) (characters 14, 23, 28, 29, 30, 32, 35, 100, 101,102,103, 107, 108,110, 111, 137) and *Porolepis* (Jarvik 1972; Jarvik 1980; Clément 2004) (characters 162, 187, 189, 191, 193, 194, 197, 199, 207, 211) have been added.

**Part II. Taxa and principal sources of data**

Osteostraci: Janvier 1981, 1985; Janvier et al. 2004;

Galeaspida: Halstead 1979; Janvier 1981; Wang 1991; Pan 1992; Gai et al. 2011;

*Yunnanolepis*: Zhang 1980; Zhu 1996; Giles et al. 2013;

*Parayunnanolepis*: Zhang et al. 2001; Zhu et al. 2012b;

*Sinolepis*: Liu and P'an 1958; Ritchie et al. 1992;

*Microbrachius*: Hemmings 1978; Long et al. 2015;

*Bothriolepis*: Young 1984; Janvier 1996; Arsenault et al. 2004; Downs and Donoghue 2009;

*Pterichthyodes*: Hemmings 1978;

*Remigolepis*: Denison 1978; Pan et al. 1980, 1987; Johanson 1997;

*Diandongpetalichthys*: P'an and Wang 1978; Zhu 1991;

*Quasipetalichthys*: Liu 1973;

*Eurycaraspis*: Liu 1991;

*Lunaspis*: Heintz 1937; Gross 1961;

*Macropetalichthys*: Stensiö 1925, 1969; Gross 1935; Denison 1978; Young 1978;

*Wuttagoonaspis*: Ritchie 1973; Young and Goujet 2003;

*Groenlandaspis*: Ritchie 1975; Anderson et al. 1994;

*Gavinaspis*: Dupret and Zhu 2008;

*Cowralepis*: Ritchie 2005; Carr et al. 2009; Long et al. 2009;

*Sigaspis*: Goujet 1973;

*Dicksonosteus*: Goujet 1975, 1984;

*Kujdanowiaspis*: Stensiö 1969; Dupret 2010;

*Buchanosteus*: Long et al. 2014;

*Parabuchanosteus*: White and Toombs 1972; White 1978; Young 1979; Long, et al. 2014;

*Holonema*: Miles 1971; Denison 1978; Trinajstic et al. 2014;

*Coccosteus*: Gross 1935; Stensiö 1963; Miles and Westoll 1968;

*Incisoscutum*: Dennis and Miles 1981; Johanson and Smith 2005; Long, et al. 2009; Giles, et al. 2013;

*Eastmanosteus*: Dennis: Bryan 1987;

*Compagopiscis*: Gardiner and Miles 1994; Trinajstic, et al. 2014;

*Materpiscis*: Long et al. 2008;

*Austroptyctodus*: Miles and Young 1977; Long 1997;{!!! INVALID CITATION !!!, ;Miles, 1977 #16791}

*Campbellodus*: Miles and Young 1977; Long 1997;

*Rhamphodopsis*: Watson 1938; Miles 1967; Miles and Young 1977; Long 1997;

*Brindabellaspis*: Young 1980, 1986; Burrow and Turner 1998, 1999; Goujet and Young 2004;

*Romundina*: Ørvig 1975; Dupret et al. 2014;

*Jagorina*: Stensiö 1969;

*Gemuendina*: Gross 1963; Young 1986;

*Entelognathus*: Zhu et al. 2013;

*Janusiscus*: Giles et al. 2015;

*Ramirosuarezia*: Pradel et al. 2009;

*Acanthodes*: Miles 1968, 1973a, b; Jarvik 1977; Denison 1979; Coates and Davis 2010; Davis et al. 2012; Brazeau and de Winter 2015

*Brachyacanthus*: Watson 1937; Denison 1979;

*Brochoadmones*: Bernacsek and Dineley 1977; Gagnier and Wilson 1996b; Hanke and Wilson 2006;

*Cassidiceps*: Gagnier and Wilson 1996a;

*Cheiracanthus*: Watson 1937; Miles 1973a; Denison 1979;

*Climatius*: Watson 1937; Ørvig 1967b; Miles 1973b, a;, , ,

*Culmacanthus*: Long 1983; Young 1989; Burrow and Young 2012;

*Diplacanthus*: Watson 1937; Miles 1973b; Denison 1979;, ,

*Euthacanthus*: Watson 1937; Miles 1973a; Newman et al. 2011;

*Gladiobranchus*: Bernacsek and Dineley 1977; Hanke and Davis 2008;

*Gyracanthides*: Miles 1973a; Warren et al. 2000; Turner et al. 2005;

*Homalacanthus*: Watson 1937; Gagnier 1996;

*Ischnacanthus*: Watson 1937; Miles 1973a; Hermus 2003;

*Kathemacanthus*: Gagnier and Wilson 1996a; Hanke and Wilson 2010;

*Latviacanthus*: Schultze and Zidek 1982;

*Lupopsyrus*: Bernacsek and Dineley 1977; Hanke and Davis 2012;

*Mesacanthus*: Watson 1937; Miles 1973a;

*Obtusacanthus*: Hanke and Wilson 2004;

*Parexus*: Watson 1937; Miles 1973a;

*Poracanthodes*: Denison 1979; Valiukevicius 1992;,

*Promesacanthus*: Hanke 2008;

*Ptomacanthus*: Miles 1973a, b; Brazeau 2009;, ,

*Rhadinacanthus*: Traquair 1888; Miles 1973a;

*Tetanopsyrus*: Gagnier 1995; Gagnier et al. 1999; Hanke et al. 2001;

*Vernicomacanthus*: Miles 1973a;

*Akmonistion*: Coates and Sequeira 1998, 2001a, b; Coates et al. 1998;

*Chondrenchelys*: Moy-Thomas 1935;

*Cladodoides*: Gross 1937, 1938; Maisey 2005;, ,

*Cladoselache*: Woodward and White 1938; Bendix: Almgreen 1975; Schaeffer 1981; Maisey 1989b, 2007;

*Cobelodus*: Zangerl and Case 1976; Maisey 2007;

*Debeerius*: Grogan and Lund 2000;

*Doliodus*: Miller et al. 2003; Maisey et al. 2009;,

*Hamiltonichthys*: Maisey 1989a;

*Helodus*: J. A. Moy: Thomas 1936;

*Onychoselache*: Dick and Maisey 1980; Maisey 1980; Coates and Gess 2007;

*Orthacanthus*: Heidtke 1982, 1998; Soler: Gijón 1999;

*Pucapampella*: Maisey 2001;

*Tamiobatis*: Romer 1964; Schaeffer 1981; Williams 1998;

*Tristychius*: Woodward 1924; Coates and Gess 2007;

*Lophosteus*: Gross 1969, 1971; Otto 1991; Burrow 1995; Schultze and Märss 2004; Botella et al. 2007;

*Dialipina*: Schultze 1968, 1992; Schultze and Cumbaa 2001;

*Ligulalepis*: Schultze 1968; Burrow 1994; Basden et al. 2000; Basden and Young 2001;,

*Cheirolepis*: Ørvig 1967a; Pearson and Westoll 1979; Pearson 1982; Arratia and Cloutier 1996, 2004;

*Howqualepis*: Long 1988;

*Mimipiscis*: Gardiner 1984; Choo 2011;,

*Moythomasia*: Gardiner 1984;

*Kentuckia*: Rayner 1951;

*Osorioichthys*: Taverne 1997;

*Meemannia*: Zhu et al. 2006, 2010; Lu et al. 2016

*Guiyu*: Zhu et al. 2009, 2012a; Qiao and Zhu 2010;

*Psarolepis*: Zhu and Schultze 1997, 2001; Yu 1998; Zhu et al. 1999; Zhu and Yu 2004, 2009; Qu et al. 2010, 2013, 2015;

*Achoania*: Zhu et al. 2001; Zhu and Yu 2004, 2009;

*Onychodus*: Jessen 1966; Long 2001; Andrews et al. 2006;

*Miguashaia*: Schultze 1973; Cloutier 1996; Forey 1998; Forey et al. 2000;

*Styloichthys*: Zhu and Yu 2002, 2004;

*Diabolepis*: Chang and Yu 1984; Chang 1995;

*Youngolepis*: Chang and Yu 1981; Chang 1982, 1991, 2004; Chang and Smith 1992;

*Powichthys*: Jessen 1975, 1980; Clément and Janvier 2004; Clément and Ahlberg 2010;

*Porolepis*: Jarvik 1972, 1980; Clément 2004;

*Glyptolepis*: Andrews and Westoll 1970; Jarvik 1972; Ahlberg 1989; Cloutier and Ahlberg 1996;

*Kenichthys*: Chang and Zhu 1993; Zhu and Ahlberg 2004;

*Osteolepis*: Jarvik 1948,1980;

*Gogonasus*: Long 1985; Long et al. 1997, 2006; Holland and Long 2009;

*Eusthenopteron*: Jarvik 1980.

**Part III. Character List:**

“ZHU”= taken from Zhu et al. 2013; “DUPRET”= taken from Dupret et al. 2014; “LONG”= taken from Long et al. 2015; “GFB”=taken from Giles et al. 2015; “BW”= taken from Brazeau and de Winter 2015; “LU” =taken from Lu et al. 2016

1. ZHU1 Tessellate prismatic calcified cartilage

0 absent

1 present

2. ZHU2 Perichondral bone

0 present

1 absent

3. ZHU3 Extensive endochondral ossification

0 absent

1 present

4. ZHU4 Dentine

0 absent

1 present

5. ZHU5 Dentine kind

0 mesodentine

1 semidentine

2 orthodentine

6. ZHU7 Lepidotrichia or lepidotrichia-like scale alignment

0 present

1 absent

7. ZHU8 Body scale growth pattern

0 monodontode

1 polyodontode

8. ZHU9 Body scale growth concentric

0 absent

1 present

9. ZHU10 Body scales with peg-and-socket articulation

0 absent

1 present

10. ZHU11 Body scale profile

0 distinct crown and base demarcated by a constriction (neck)

1 flattened

11. ZHU12 Body scales with bulging base

0 absent

1 present

12. ZHU13 Body scales with flattened base

0 present

1 absent

13. ZHU14 Flank scales alignment

0 vertical rows

1 oblique rows or hexagonal/rhombic packing

2 disorganised

14. ZHU15 Sensory line canal

0 passes between or beneath scales

1 passes over scales and/or is partially enclosed or surrounded by scales

2 perforates and passes through scales

15. ZHU16 Sensory line network

0 preserved as open grooves

1 pass through canals enclosed within dermal bones

16. ZHU17 Jugal portion of infraorbital canal joins supramaxillary canal

0 present

1 absent

17. ZHU18 Dermal skull roof

0 includes large dermal plates

1 consists of undifferentiated plates or tesserae

18. ZHU19 Tesserae morphology

0 large interlocking polygonal plates

1 microsquamose, not larger than body tesserae

19. ZHU20 Extent of dermatocranial cover

0 complete

1 incomplete (scale-free and elsewhere)

20. ZHU21 Endolymphatic ducts open in dermal skull roof

0 present

1 absent

21. ZHU22 Endolymphatic ducts with oblique course through dermal skull bones

0 absent

1 present

22. ZHU23 Series of paired median skull roofing bones that meet at the dorsal midline of the skull (rectilinear skull roof pattern)

0 absent

1 present

23. ZHU24 Consolidated cheek plates

0 absent

1 present

24. ZHU25 Pineal opening perforation in dermal skull roof

0 present

1 absent

25. ZHU26 Enlarged postorbital tessera separate from orbital series

0 absent

1 present

26. ZHU27 Bony hyoidean gill-cover series (branchiostegals)

0 absent

1 present

27. ZHU28 Branchiostegal plate series along ventral margin of lower jaw

0 absent

1 present

28. ZHU29 Branchiostegal ossifications

0 plate-like

1 narrow and ribbon-like

29. ZHU30 Branchiostegal ossifications

0 ornamented

1 unornamented

30. ZHU31 Imbricated branchiostegal ossifications

0 absent

1 present

31. ZHU32 Opercular cover of branchial chamber

0 complete or partial

1 separate gill covers and gill slits

32. ZHU33 Opercular (submarginal) ossification

0 absent

1 present

33. ZHU34 Shape of opercular (submarginal) ossification

0 broad plate that tapers towards its proximal end

1 narrow, rod-shaped

34. ZHU35 Gular plates

0 absent

1 present

35. ZHU36 Size of lateral gular plates

0 extending most of length of the lower jaw

1 restricted to theanterior third of the jaw (no longer than the width of three or four branchiostegals

36. ZHU37 Basihyal

0 present

1 absent, hyoid arch articulates directly with basibranchial

37. ZHU38 Interhyal

0 absent

1 present

38. ZHU39 Oral dermal tubercles borne on jaw cartilages

0 absent

1 present

39. ZHU40 Tooth whorls

0 absent

1 present

40. ZHU41 Bases of tooth whorls

0 single, continuous plate

1 some or all whorls consist of separate tooth units

41. ZHU42 Enlarged adsymphysial tooth whorl

0 absent

1 present

42. ZHU43 Teeth ankylosed to dermal bones

0 absent

1 present

43. ZHU44 Dermal jaw plates on biting surface of jaw cartilages

0 absent

1 present

44. ZHU45 Maxillary and dentary tooth-bearing bones

0 absent

1 present

45. ZHU46 Large otic process of the palatoquadrate

0 absent

1 present

46. ZHU47 Insertion area for jaw adductor muscles on palatoquadrate

0 ventral

1 lateral

47. ZHU48 Oblique ridge or groove along medial face of palatoquadrate

0 absent

1 present

48. ZHU49 Fenestration of palatoquadrate at basipterygoid articulation

0 absent

1 present

49. ZHU50 Perforate or fenestrate anterodorsal (metapterygoid) portion of palatoquadrate

0 absent

1 present

50. ZHU51 Pronounced dorsal process on Meckelian bone or cartilage

0 absent

1 present

51. ZHU52 Preglenoid process

0 absent

1 present

52. ZHU53 Jaw articulation located on rearmost extremity of mandible

0 absent

1 present

53. ZHU54 Precerebral fontanelle

0 absent

1 present

54. ZHU55 Median dermal bone of palate (parasphenoid)

0 absent

1 present

55. ZHU56 Nasal opening(s)

0 dorsal, placed between orbits

1 ventral and anterior to orbits

56. ZHU57 Olfactory tracts

0 short, with olfactory capsules situated close to telencephalon cavity

1 elongate and tubular (much longer than wide)

57. ZHU58 Prominent pre-orbital rostral expansion of the neurocranium

0 present

1 absent

58. ZHU59 Pronounced sub-ethmoidal keel

0 absent

1 present

59. ZHU60 Position of myodome for superior oblique eye muscles

0 posterior and dorsal to foramen for nerve II

1 anterior and dorsal to foramen

60. ZHU61 Endoskeletal cranial joint

0 absent

1 present

61. ZHU62 Spiracular groove on basicranial surface

0 absent

1 present

62. ZHU63 Spiracular groove on lateral commissure

0 absent

1 present

63. ZHU64 Subpituitary fenestra

0 absent

1 present

64. ZHU65 Supraorbital shelf broad with convex lateral margin

0 absent

1 present

65. ZHU66 Orbit dorsal or facing dorsolaterally, surrounded laterally by endocranium

0 present

1 absent

66. ZHU67 Extended prehypophysial portion of sphenoid

0 absent

1 present

67. ZHU68 Narrow interorbital septum

0 absent

1 present

68. ZHU69 Main trunk of facial nerve (N. VII)

0 is elongate and passes anterolaterally through orbital floor

1 is stout and divides within otic capsule at the level of the postorbital process

69. ZHU70 Hyoid ramus of facial nerve (N. VII) exits through posterior jugular opening

0 absent

1 present

70. ZHU71 Glossopharyngeal nerve (N. IX) exit

0 foramen situated posteroventral to otic capsule and anterior to metotic fissure

1 through metotic fissure

71. ZHU72 Short otico-occipital region of braincase

0 absent

1 present

72. ZHU73 Ethmoid region elongate with dorsoventrally deep lateral walls

0 absent

1 present

73. ZHU74 Basicranial morphology

0 platybasic

1 tropibasic

74. ZHU75 Ascending basisphenoid pillar pierced by common internal carotid

0 absent

1 present

75. ZHU79 Canal for efferent pseudobranchial artery within basicranial cartilage

0 absent

1 present

76. ZHU95 Position of hyomandibula articulation on neurocranium

0 absent

1 present

77. ZHU77 Canal for lateral dorsal aorta within basicranial cartilage

0 absent

1 present

78. ZHU78 Entrance of internal carotids

0 through separate openings flanking the hypophyseal opening or recess

1 through a common opening at the central midline of the basicranium

79. ZHU80 Position of basal/basipterygoid articulation

0 same anteroposterior level as hypophysial opening

1 anterior to hypophysial opening

80. ZHU81 Postorbital process articulates with palatoquadrate

0 absent

1 present

81. ZHU82 Labyrinth cavity

0 separated from the main neurocranial cavity by a cartilaginous or ossified capsular wall

1 skeletal capsular wall absent

82. ZHU83 Basipterygoid process (basal articulation) with vertically oriented component

0 absent

1 present

83. ZHU84 Pituitary vein canal

0 dorsal to level of basipterygoid process

1 flanked posteriorly by basipterygoid process

84. ZHU85 External (horizontal) semicircular canal

0 absent

1 present

85. ZHU86 Sinus superior

0 absent or indistinguishable from union of anterior and posterior canals with saccular chamber

1 present

86. ZHU87 External (horizontal) semicircular canal

0 joins the vestibular region dorsal to posterior ampulla

1 joins level with posterior ampulla

87. ZHU88 Trigemino-facial recess

0 absent

1 present

88. ZHU89 Posterior dorsal fontanelle

0 absent

1 present

89. ZHU90 Shape of posterior dorsal fontanelle

0 approximately as long as broad

1 much longer than wide, slot-shaped

90. ZHU91 Dorsal ridge

0 absent

1 present

91. ZHU92 Endolymphatic ducts

0 posteriodorsally angled tubes

1 tubes oriented vertically through median endolymphatic fossa

92. ZHU96 Ventral cranial fissure

0 absent

1 present

93. ZHU97 Metotic (otic-occipital) fissure

0 absent

1 present

94. ZHU98 Vestibular fontanelle

0 absent

1 present

95. ZHU99 Occipital arch wedged in between otic capsules

0 absent

1 present

96. ZHU100 Spino-occipital nerve foramina

0 two or more, aligned horizontally

1 one or two, dorsoventrally offset

97. ZHU101 Ventral notch between parachordals

0 absent

1 present or entirely unfused

98. ZHU102 Parachordal shape

0 broad, flat

1 keeled with sloping lateral margins

99. ZHU103 Hypotic lamina (and dorsally directed glossopharyngeal canal)

0 absent

1 present

100. ZHU104 Macromeric dermal shoulder girdle

0 present

1 absent

101. ZHU105 Dermal shoulder girdle composition

0 ventral and dorsal (scapular) components

1 ventral components only

102. ZHU106 Dermal shoulder girdle forming a complete ring around the trunk

0 present

1 absent

103. ZHU107 Pectoral fenestra completely encircled by dermal shoulder armour

0 present

1 absent

104. ZHU108 Median dorsal plate

0 absent

1 present

105. ZHU109 Pronounced internal crista (keel) on median dorsal surface of shoulder girdle

0 absent

1 present

106. ZHU110 Scapular process of shoulder endoskeleton

0 absent

1 present

107. ZHU111 Ventral margin of separate scapular ossification

0 horizontal

1 deeply angled

108. ZHU112 Cross sectional shape of scapular process

0 flattened or strongly ovate

1 subcircular

109. ZHU113 Flange on trailing edge of scapulocoracoid

0 absent

1 present

110. ZHU114 Scapular process with posterodorsal angle

0 absent

1 present

111. ZHU115 Endoskeletal postbranchial lamina on scapular process

0 present

1 absent

112. ZHU116 Mineralisation of internal surface of scapular blade

0 mineralised all around

1 unmineralised on internal face forming a hemicylindrical cross-section

113. ZHU117 Coracoid process

0 absent

1 present

114. ZHU118 Procoracoid mineralisation

0 absent

1 present

115. ZHU119 Fin base articulation on scapulocoracoid

0 stenobasal

1 eurybasal

116. ZHU120 Perforate propterygium

0 absent

1 present

117. ZHU121 Pelvic fins

0 absent

1 present

118. ZHU122 Intromittent organ containing bone, not associated with pelvic fins

0 absent

1 present

119. ZHU123 Dermal pelvic clasper ossifications

0 absent

1 present

120. ZHU124 Pectoral fins covered in macromeric dermal armour

0 absent

1 present

121. ZHU125 Pectoral fin base has large, hemispherical dermal component

0 absent

1 present

122. ZHU126 Dorsal fin spines

0 absent

1 present

123. ZHU127 Anal fin spine

0 absent

1 present

124. ZHU128 Paired fin spines

0 absent

1 present

125. ZHU129 Median fin spine insertion

0 shallow, not greatly deeper than dermal bones / scales

1 deep

126. ZHU130 Intermediate fin spines

0 absent

1 present

127. ZHU131 Prepectoral fin spines

0 absent

1 present

128. ZHU132 Fin spines with ridges

0 absent

1 present

129. ZHU133 Fin spines with nodes

0 absent

1 present

130. ZHU134 Fin spines with rows of large retrorse denticles

0 absent

1 present

131. ZHU135 Synarcual

0 absent

1 present

132. ZHU136 Number of dorsal fins, if present

0 one

1 two

133. ZHU137 Anal fin

0 absent

1 present

134. ZHU138 Caudal radials

0 extend beyond level of body wall and deep into hypochordal lobe

1 restricted to axial lobe

135. ZHU196, GFB67 Median gular

0 present

1 absent

136. ZHU140, GFB80 Acrodin

0 absent

1 present

137. ZHU141, GFB86 Plicidentine

0 absent

1 simple or generalized polyplacodont

138. ZHU143, DFB19 Peg on rhomboid scale

0 narrow

1 broad

139. ZHU144, GFB20 Anterodorsal process on scale

0 absent

1 present

140. ZHU146, GFB236 Epichordal lepidotrichia in caudal fin

0 absent

1 present

141. ZHU147, GFB46 Dermal intracranial joint

0 absent

1 present

142. ZHU152, GFB116 Posterior nostril

0 associated with orbit

1 not associated with orbit

143. ZHU161, GFB48 Number of marginal bones alongside paired median skull roofing bones over the otico-occipital division of braincase

0 single

1 two or more

144. ZHU169, GFB60 Type of dermal neck-joint

0 sliding, dermal shoulder girdle plate with flat articular flange

1 ginglymoid, dermal shoulder girdle plate with articular condyle

2 reversed ginglymoid,dermal shoulder girdle plate with articular fossa

3 spoon-like

4 simple contact

145. ZHU180, GFB90 Posterior expansion of maxilla (maxilla cleaver-shaped)

0 present

1 absent

146. ZHU182, GFB59 Contribution by maxilla to posterior margin of cheek

0 present

1 absent

147. ZHU201, GFB106 Number of coronoids

0 more than three

1 three

148. ZHU202, GFB94 Fangs of coronoids (sensu stricto)

0 absent

1 present

149. ZHU222, GFB131 Eye stalk or unfinished area on neurocranial wall for eye stalk

0 absent

1 present

150. ZHU227, GFB155 Articulation facet with hyomandibular

0 single-headed

1 double-headed

151. ZHU231, GFB169 Basicranial fenestra

0 absent

1 present

152. ZHU233, GFB155 Lateral cranial canal

0 absent

1 present

153. ZHU234, GFB179 Midline canal in basicranium for dorsal aorta

0 absent

1 present

154. ZHU239, GFB113 Ascending process of parasphenoid

0 absent

1 present

155. ZHU240, GFB111 Shape of parasphenoid denticulated field

0 broad rhomboid or lozenge-shaped

1 broad, splint-shaped

2 slender, splint-shaped

156. ZHU241, GFB112 Parasphenoid denticulated field with multifid anterior margin

0 absent

1 present

157. ZHU139 Resorption and redeposition of odontodes

0 lacking or partially developed

1 developed

158. ZHU142 Rostral tubuli

0 absent

1 present

159. ZHU148 Large unpaired median skull roofing bone anterior to the level of nasal capsules

0 absent

1 present

160. ZHU149 Number of nasals

0 many

1 one or two

161. ZHU150 Mesial margin of nasal

0 not notched

1 notched

162. ZHU151 Dermintermedial process

0 absent

1 present

163. ZHU153 Position of posterior nostril

0 external, far from jaw margin

1 external, close to jaw margin

164. ZHU154 Supraorbital (sensu Cloutier and Ahlberg 1996, including posterior tectal of Jarvik)

0 absent

1 present

165. ZHU155 Supraorbital, preorbital and nasal

0 unfused

1 fused

166. ZHU156 Tectal (sensu Cloutier and Ahlberg 1996, not counting the posterior tectal of Jarvik)

0 absent

1 present

167. ZHU157 Lateral plate

0 absent

1 present

168. ZHU158 Location of pineal foramen/eminence

0 level with posterior margin of orbits

1 well posterior of orbits

169. ZHU159 Parietals (preorbitals of placoderms) surround pineal foramen or eminence

0 yes

1 no

170. ZHU160 Complete enclosure of spiracle by skull roof bones

0 absent

1 present

171. ZHU162 paranuchal number

0 one pair

1 two pairs

172. ZHU163 Large unpaired median bone contributing to posterior margin of skull roof

0 absent

1 present

173. ZHU164 Contact of nuchal or centronuchal plate with paired preorbital plates

0 absent

1 present

174. ZHU165 Posterior process of the paranuchal plate behind the nuchal plate (dorsal face)

0 absent

1 present

175. ZHU166 Junction of posterior pitline and main lateral line

0 far in front of posterior margin of skull roof

1 close to posterior margin of skull roof

176. ZHU167 Number of extrascapulars

0 uneven

1 paired

177. ZHU168 Dermal neck-joint between paired main-lateral-line-bearing bones of skull and shoulder girdle

0 absent

1 present

178. ZHU171 Foramina (similar to infradentary foramina) on cheek bones

0 absent

1 present

179. ZHU172 Lacrimal posteriorly enclosing posterior nostril

0 absent

1 present

180. ZHU173 Most posterior major bone of cheek bearing preopercular canal (preopercular) extending forward, close to orbit

0 absent

1 present

181. ZHU174 Number of cheek bones bearing preopercular canal posterior to jugal

0 one

1 two

182. ZHU175 Bone bearing both quadratojugal pit-line and preopercular canal

0 absent

1 present

183. ZHU176 Dermohyal

0 absent

1 present

184. ZHU177 Premaxillae with inturned symphysial processes

0 absent

1 present

185. ZHU178 Premaxilla forming part of orbit

0 absent

1 present

186. ZHU179 Preorbital process of premaxilla

0 absent

1 present

187. ZHU181 Ventral margin of maxilla

0 straight

1 curved

188. ZHU183 Course of ethmoid commissure

0 middle portion through median rostral

1 sutural course

2 through bone center of premaxillary

189. ZHU184 Position of anterior pit-line

0 on paired median skull roofing bones over the otico-occipital division of braincase

1 on paired median skull roofing bones over the sphenoid division of braincase

190. ZHU185 Middle and posterior pit-lines on postparietal

0 posteriorly situated

1 mesially situated

191. ZHU186 Position of middle and posterior pit lines

0 close to midline

1 near the central portion of each postparietal

192. ZHU187 Course of supraorbital canal

0 between anterior and posterior nostrils

1 anterior to both nostrils

193. ZHU188 Course of supraorbital canal

0 straight

1 lyre-shaped

194. ZHU189 Posterior end of supraorbital canal

0 in postparietal

1 in parietal

2 in intertemporal

3 in nuchal plate

4 in postpineal plate

195. ZHU190 Contact between otic and supraorbital canals

0 not in contact

1 in contact

196. ZHU191 Contact of supraorbital and infraorbital canals

0 in contact rostrally

1 not in contact rostrally

197. ZHU192 Otic canal

0 runs through skull roof

1 follows edge of skull roof

198. ZHU193 Infraorbital canal follows premaxillary suture

0 no

1 yes

199. ZHU194 Sensory canal or pit-line associated with maxilla

0 absent

1 present

200. ZHU195 Anterior portion of preopercular canal

0 present

1 absent

201. ZHU197 Foramen in hyomandibular

0 absent

1 present

202. ZHU198 Large dermal plates forming outer dental arcade

0 only with denticles

1 with large monolinear tooth row

203. ZHU199 Tooth-bearing median rostral

0 absent

1 present

204. ZHU200 Teeth of dentary

0 reaching anterior end of dentary

1 not reaching anterior end

205. ZHU203 Marginal denticle band on coronoids

0 broad band, at least posteriorly

1 narrow band with 2-4 denticle rows

206. ZHU204 infradentary

0 absent

1 present

207. ZHU205 Infradentary foramina

0 present

1 absent

208. ZHU206 Large ventromesially directed flange of symphysial region of mandible

0 absent

1 present

209. ZHU207 Flange like extension of mandible composed of prearticular and Meckelian ossification

0 absent

1 present

210. ZHU208 Strong ascending flexion of symphysial region of mandible

0 absent

1 present

211. ZHU209 Parasymphysial plate

0 detachable tooth whorl

1 long with posterior corner, sutured to coronoid, denticulated or with tooth row

2 absent

212. ZHU210 Anterior end of prearticular

0 far from jaw symphysis

1 near jaw symphysis

213. ZHU211 Prearticular - dentary contact

0 present

1 absent

214. ZHU212 Meckelian bone exposed immediately anterior to first coronoid

0 yes

1 no

215. ZHU213 Dermal plates on mesial (lingual) surfaces of Meckels cartilage and palatoquadrate

0 absent

1 present

216. ZHU214 Biconcave glenoid on lower jaw

0 absent

1 present

217. ZHU217 Course of mandibular canal

0 not passing through most posterior infradentary

1 passing through most posterior infradentary

218. ZHU218 Course of mandibular canal

0 passing through dentary

1 not passing through dentary

219. ZHU220 Fenestra ventrolateralis

0 absent

1 present

2 common ventral fenestra for anterior and posterior nostrils

220. ZHU223 Developed postorbital cavity

0 absent

1 present

221. ZHU225 Unconstricted cranial notochord

0 absent

1 present

222. ZHU226 Descending process of sphenoid (with its posterior extremity lacking periostegeal lining)

0 absent

1 present

223. ZHU228 Hyoid arch articulation

0 on lateral commissure

1 on otic capsule wall

224. ZHU229 Opercular suspension on braincase

0 absent

1 present

225. ZHU235 Vomerine fangs

0 absent

1 present

226. ZHU236 Vomeral area with grooves and raised areas

0 absent

1 present

227. ZHU237 Parasphenoid

0 protruding forward into ethmoid region of endocranium

1 behind ethmoid region

228. ZHU238 Denticulated field of parasphenoid

0 without spiracular groove

1 with spiracular groove

229. ZHU242 Parasphenoid denticle field with anteriorly divergent lateral margins

0 absent

1 present

230. ZHU243 Parasphenoid denticle field

0 terminates at or anterior to level of foramina for internal carotid arteries

1 extends posterior to foramina for internal carotid arteries

231. ZHU244 Presupracleithrum

0 absent

1 present

232. ZHU245 Anocleithrum

0 element developed as postcleithrum

1 element developed as anocleithrum sensu stricto

233. ZHU250, GFB201 Endoskeletal supports in pectoral fin

0 multiple elements articulating with girdle

1 single element ("humerus") articulating with girdle

234. ZHU246 Dorsal cleithrum (AL of the Placodermi), ventral cleithrum (AVL of the Placodermi) and pectoral spine (SP of the Placodermi)

0 not fused

1 fused

235. ZHU247 Relationship of clavicle to cleithrum

0 ascending process of clavicle overlapping cleithrum laterally

1 ascending process of clavicle wrapping round anterior edge of cleithrum, overlapping it both laterally and mesially

236. ZHU248 Triradiate scapulocoracoid

0 absent

1 present

237. ZHU249 Subscapular foramen

0 absent

1 present

238. ZHU251 Pectoral propterygium

0 absent

1 present

239. ZHU252 Pelvic girdle with substantial dermal component

0 yes

1 no

240. ZHU253 Pelvic fin spine

0 absent

1 present

241. ZHU170 Number of sclerotic plates

0 four or less

1 more than four

242. ZHU221 Ethmoid articulation for palatoquadrate

0 placed on postnasal wall

1 extends posteriorly to the level of N.II

243. ZHU215 Contact between palatoquadrate and dermal cheek bones

0 continuous contact of metapterygoid and autopalatine

1 metapterygoid and autopalatine contacts separated by gap between commissural lamina of palatoquadrate and cheek bones

244. ZHU216 Metapterygoid with developed medial ventral protrusion

0 absent

1 present

245. ZHU219 Internasal pits

0 absent

1 undifferentiated or anterior palatal fossa

2 shallow, paired pits with strong midline ridge

3 deep, peer-shaped pits

246. DUPRET254 jaws

0 absent

1 present

247. DUPRET255, GFB121 optic fissure

0 present

1 absent

248. LONG256 Central dermal skull bone (nuchal) with converging posterior pit-line canals and supraorbital canals

0 absent

1 converging but not meeting

2 crossing as an X in bone

249. LONG257 Deep, high supragnathal bone with durophagous occlusal surface

0 absent

1 present

250. LONG258 Intromittent organ with one large J-shaped element

0 absent

1 present

251. LONG259 Intromittent organ ('clasper') consisting entirely of cartilage, formed from distal part of pelvic fin

0 absent

1 present

252. ZHU145 Fringing fulcra

0 absent

1 present

253. GFB2 Prismatic calcified cartilage

0 single layered

1 multi-layered

254. GFB5 Enamel(oid) present on dermal bones and scales

0 absent

1 present

255. GFB6 Enamel

0 single-layered

1 multi-layered

256. GFB7 Enamel layers

0 applied directly to one another (ganoine)

1 separated by layers of dentine

257. GFB8 Extensive pore canal network

0 absent

1 present

258. GFB57 Vertical canal associated with preopercular/suborbital canal

0 absent

1 present

259. GFB11 Bone cell lacunae in body scale bases

0 present

1 absent

260. GFB12 Main dentinous tissue forming fin spine

0 osteodentine

1 orthodentine

261. GFB14 Differentiated lepidotrichia

0 absent

1 present

262. GFB22 Profile of scales with constriction between crown and base

0 neck similar in width to crown

1 neck greatly constricted, resulting in anvil-like shape

263. GFB25 Basal pore in scales

0 absent

1 present

264. GFB27 Scute-like ridge scales (basal fulcra)

0 absent

1 present

265. GFB29 Dermal ornamentation

0 smooth

1 parallel, vermiform ridges

2 concentric ridges

3 tuberculate

266. GFB31 Sensory canals/grooves

0 contained within the thickness of dermal bones

1 contained in prominent ridges on visceral surface of bone

267. GFB34 Anterior pit line of dermal skull roof

0 absent

1 present

268. GFB36 Cranial spines

0 absent

1 present, multicuspid

2 present, monocuspid

269. GFB40 Endolymphatic duct relationship to median skull roof bone (i.e. nuchal plate)

0 within median bone

1 on bones flanking the median bone (e.g. paranuchals)

270. GFB42 Dermal plate associated with pineal eminence or foramen

0 contributes to orbital margin

1 plate bordered laterally by skull roofing bones

271. GFB44 Broad supraorbital vaults

0 absent

1 present

272. GFB45 Median commisure between supraorbital sensory lines

0 absent

1 present

273. GFB47 Otic canal extends through postparietals

0 absent

1 present

274. GFB49 Suture between paired skull roofing bones (centrals of placoderms; postparietals of osteichthyans)

0 straight

1 sinusoidal

275. GFB50 Medial processes of paranuchal wrapping posterolateral corners of nuchal plate

0 absent

1 present

2 paranuchals precluded from nuchal by centrals

3 no median posterior skull roof bone

276. GFB51 Paired pits on ventral surface of nuchal plate

0 absent

1 present

277. GFB52 Sclerotic ring

0 absent

1 present

278. GFB54 Cheek plate

0 undivided

1 divided (i.e., squamosal and preopercular)

279. GFB55 Subsquamosals in taxa with divided cheek

0 absent

1 present

280. GFB56 Preopercular shape

0 rhombic

1 bar-shaped

281. GFB72 Gill arches

0 largely restricted to region under braincase

1 extend far posterior to braincase

282. GFB75 Hypohyal

0 absent

1 present

283. GFB76 Endoskeletal urohyal

0 absent

1 present

284. GFB79 Enamel(oid) on teeth

0 absent

1 present

285. GFB84 Distribution of tooth whorls

0 upper and lower jaws

1 lower jaws only

2 upper jaws only

286. GFB89 Premaxilla

0 extends under orbit

1 restricted anterior to orbit

287. GFB91 Pair of tooth plates (anterior supragnathals or vomers) on ethmoidal plate

0 absent

1 present

288. GFB93 Extent of infradentaries

0 along much of ventral margin of dentary

1 restricted to posterior half of dentary

289. GFB95 Position of upper mandibular arch cartilage (and associated cheek plate where present)

0 entirely suborbital

1 with a postorbital extension

290. GFB97 Autopalatine and quadrate

0 comineralized

1 separate mineralizations

291. GFB101 Palatoquadrate fused with neurocranium

0 absent

1 present

292. GFB114 Buccohypophysial canal in parasphenoid

0 single

1 paired

293. GFB125 Transverse otic process

0 present

1 absent

294. GFB126 Jugular canal

0 long (invested in otic region along length of skeletal labyrinth)

1 short (restricted to region anterior of skeletal labyrinth)

2 absent (jugular vein uninvested in otic region)

295. GFB132 Postorbital process

0 absent

1 present

296. GFB133 Canal for jugular in postorbital process

0 absent

1 present

297. GFB134 Series of perforations for innervation of supraorbital sensory canal in supraorbital shelf

0 absent

1 present

298. GFB141 Subcranial ridges

0 absent

1 present

299. GFB154 Horizontal semicircular canal in dorsal view

0 medial to path of jugular vein

1 dorsal to jugular vein

300. GFB159 Synotic tectum

0 absent

1 present

301. GFB161 Shape of median dorsal ridge anterior to endolymphatic fossa

0 developed as a squared-off ridge or otherwise ungrooved

1 bears a midline groove

302. GFB166 Branchial ridges

0 present

1 reduced to vagal process

2 absent (articulation made with bare cranial wall)

303. GFB167 Craniospinal process ("supravagal process" in Stensio)

0 absent

1 present

304. GFB176 Stalk-shaped parachordal/occipital region

0 absent

1 present

305. GFB177 Paired occipital facets

0 absent

1 present

306. GFB178 Size of aperture to notochordal canal

0 much smaller than foramen magnum

1 as large, or larger, than foramen magnum

307. GFB183 Shape of dorsal blade of dermal shoulder girdle

0 spatulate

1 pointed

308. GFB187 Posterior dorsolateral (PDL) plate or equivalent

0 absent

1 present

309. GFB190 Scapular infundibulum

0 absent

1 present

310. GFB202 Number of basals in polybasal pectoral fins

0 three or more

1 two

311. GFB204 Number of mesomeres in metapterygial axis

0 five or fewer

1 seven or more

312. GFB205 Biserial pectoral fin endoskeleton

0 absent

1 present

313. GFB207 Filamentous extension of pectoral fin from axillary region

0 absent

1 present

314. GFB235 Supraneurals in axial lobe of caudal fin

0 absent

1 present

315. GFB218 Fin spine cross-section

0 round or horseshoe shaped

1 Flat-sided, with rectangular profile

316. GFB219 Intermediate spines when present

0 one pair

1 multiple pairs

317. GFB224 Expanded spine rib on leading edge of spine

0 absent

1 present

318. GFB225 Spine ridges

0 converging at the distal apex of the spine

1 converging on leading edge of spine

319. GFB227 Series of thoracic supraneurals

0 absent

1 present

320. GFB229 Posterior dorsal fin shape

0 base approximately as broad as tall, not broader than all of other median fins

1 base much longer than the height of the fin, substantially longer than any of the other dorsal fins

321. GFB230 Basal plate in dorsal fin (Friedman & Brazeau (2010: character 42).)

0 absent

1 present

322. GFB231 Branching radial structure articulating with dorsal fin basal plate

0 absent

1 present

323. GFB233 Basal plate in anal fin (Friedman & Brazeau (2010: character 42).)

0 absent

1 present

324. BW237 Relative position of jugular groove and hyomandibular articulation:

0 hyomandibula dorsal or same level (i.e. on bridge)

1 jugular vein passing dorsal or lateral to hyomandibula

325. LU238 Canal-bearing bone of skull roof extends far past posterior margin of parietals.

0 no

1 yes

326. LU 240 Position of anterior pitline

0 on postparietal

1 on parietal

327. LU 241 Opening in dermal skull roof for spiracular bounded by bones carrying otic canal

0 absent

1 present

328. LU 244 Preoperculosubmandibular

0 absent

1 present

329. LU 246 Urohyal shape

0 absent

1 Vertical plate

330. LU 252 Extensive flange composed of prearticular and Meckelian bone that extends beyond ventral edge of outer dermal series

0 absent

1 present

331. LU 255 Inturned medial process of premaxilla

0 absent

1 present

332. LU 262 Posterior nostril

0 facial

1 at margin oral cavity

2 palatal

333. LU 265 Size of profundus canal in postnasal wall

0 samall

1 large

334. LU 266 Paired pineal and parapineal tracts

0 absent

1 present

335. LU 268 Endoskeletal spiracular canal

0 open

1 partial enclosure or spiracular bar

2 complete enclosure in canal

Ahlberg P.E. 1989. Paired fin skeletons and relationships of the fossil group Porolepiformes (Osteichthyes: Sarcopterygii). Zool. J. Linn. Soc. 96:119-166.

Anderson M.E., Hiller N., Gess R.W. 1994. The first *Bothriolepis*-associated Devonian fish fauna from Africa. S. Afr. J. Sci. 90:397-403.

Andrews S.M., Long J.A., Ahlberg P.E., Barwick R., Campbell K.S.W. 2006. The structure of the sarcopterygian *Onychodus jandemarrai* n. sp. from Gogo, Western Australia: with a functional interpretation of the skeleton. Trans. R. Soc. Edinb. Earth Sci. 96:197- 307.

Andrews S.M., Westoll T.S. 1970. The postcranial skeleton of rhipidistian fishes excluding Eusthenopteron. Trans. R. Soc. Edinb. Earth Sci. 68:391-486.

Arratia G., Cloutier R. 1996. Reassessment of the morphology of *Cheirolepis canadensis* (Actinopterygii). In: Schultze H.-P., Cloutier R., editors. Devonian fishes and plants of Miguasha, Quebec, Canada. München: Verlag Dr Friedrich Pfeil. p. 165-197.

Arratia G., Cloutier R. 2004. A new cheirolepidid fish from the Middle-Upper Devonian of Red Hill, Nevada, USA. In: Arratia G., Wilson M.V.H., Cloutier R., editors. Recent advances in the origin and early radiation of vertebrates. München: Verlag Dr. Friedrich Pfeil. p. 583-598.

Arsenault M., Desbiens S., Janvier P., Kerr J. 2004. New data on the soft tissues and external morphology of the antiarch *Bothriolepis canadensis* (Whiteaves, 1880) from the Upper Devonian of Miguasha, Quebec. In: Arratia G., Wilson M.V.H., Cloutier R., editors. recent advances in the origin and early radiation of vertebrates. München: Verlag Dr. Friedrich Pfeil. p. 439-454.

Basden A.M., Young G.C. 2001. A primitive actinopterygian neurocranium from the Early Devonian of southeastern Australia. J. Vert. Paleo. 21:754-766.

Basden A.M., Young G.C., Coates M.I., Ritchie A. 2000. The most primitive osteichthyan braincase? Nature 403:185-188.

Bendix-Almgreen S.E. 1975. The paired fins and shoulder girdle in Cladoselache, their morphology and phyletic significance. In: Lehman J.P., editor. Problèmes actuels de paléontologie-evolution des vertébrés. Paris: Colloques Internationaux du Centre National de la Recherche Scientifique. p. 111-123.

Bernacsek G.M., Dineley D.L. 1977. New acanthodians from the Delorme Formation (Lower Devonian) of N.W.T., Canada. Palaeontogr. Abt. A 158:1-25.

Botella H., Blom H., Dorka M., Ahlberg P.E., Janvier P. 2007. Jaws and teeth of the earliest bony fishes. Nature 448:583-586.

Brazeau M.D. 2009. The braincase and jaws of a Devonian ‘acanthodian’ and modern gnathostome origins. Nature 457:305-308.

Brazeau MD, de Winter V. 2015. The hyoid arch and braincase anatomy of *Acanthodes* support chondrichthyan affinity of 'acanthodians'. Proc R Soc B 282:20152210. doi: http://dx.doi.org/10.1098/rspb.2015.2210.

Burrow C.J. 1994. Form and function in scales of *Ligulalepis toombsi* Schultze, a palaeoniscoid from the Early Devonian of Australia. Rec. S. Aust. Mus. 27:175-185.

Burrow C.J. 1995. A new Lophosteiform (Osteichthyes) from the Lower Devonian of Australia. Geobios M. S. 19:327-333.

Burrow C.J., Turner S. 1998. Devonian placoderm scales from Australia. J. Vert. Paleo. 18:677-695.

Burrow C.J., Turner S. 1999. A review of placoderm scales, and their significance in placoderm phylogeny. J. Vert. Paleo. 19:204-219.

Burrow C.J., Young G.C. 2012. New Information on *Culmacanthus* (Acanthodii:Diplacanthiformes) from the ?Early–Middle Devonian of Southeastern Australia. Proc. Linn. Soc. NSW 134:21-29.

Carr R.K., Johanson Z., Ritchie A. 2009. The phyllolepid placoderm *Cowralepis mclachlani*: Insights into the evolution of feeding mechanisms in jawed vertebrates. J. Morp. 270:775-804.

Chang M.-M. 1982. The braincase of *Youngolepis*, a Lower Devonian crossopterygian from Yunnan, south-western China. Stockholm: University of Stockholm, Department of Geology.

Chang M.-M. 1991. Head exoskeleton and shoulder girdle of *Youngolepis*. In: Chang M.-M., Liu Y.-H., Zhang G.-R., editors. Early vertebrates and related problems of evolutionary biology. Beijing: Science Press. p. 355-378.

Chang M.-M. 1995. *Diabolepis* and its bearing on the relationships between porolepiforms and dipnoans. Bull. Mus. Nat. His. Nat. (section C) 1-4:235-268.

Chang M.-M. 2004. Synapomorphies and scenarios - more characters of *Youngolepis* betraying its affinity to the Dipnoi. In: Arratia G., Wilson M.V.H., Cloutier R., editors. Recent advances in the origin and early radiation of vertebrates. München: Verlag Dr. Friedrich Pfeil. p. 665-686.

Chang M.-M., Smith M.M. 1992. Is *Youngolepis* a porolepiform? J. Vert. Paleo. 12:294-312.

Chang M.-M., Yu X.-B. 1981. A new crossopterygian, *Youngolepis praecursor*, gen. et sp. nov., from Lower Devonian of E. Yunnan, China. Sci. Sin. 24:89-97.

Chang M.-M., Yu X.-B. 1984. Structure and phylogenetic significance of *Diabolichthys speratus* gen. et sp. nov., a new dipnoan-like form from the Lower Devonian of eastern Yunnan, China. Proc. Linn. Soc. NSW 107:171-184.

Chang M.M., Zhu M. 1993. A new osteolepidid from the Middle Devonian of Qujing, Yunnan. Mem. Ass. Aust. Palaeontols. 15:183-198.

Choo B. 2011. Revision of the actinopterygian genus *Mimipiscis* (=*Mimia*) from the Upper Devonian Gogo Formation of Western Australia and the interrelationships of the early Actinopterygii. Earth Environ. Sci. Trans. Roy. Soc. Edinb. 102:77-104.

Clément G. 2004. Nouvelles données anatomiques et morphologie générale des Porolepididae (Diplnomorpha, Sarcopterygii). Rev. Paléo. Gen. 9:193-211.

Clément G., Ahlberg P.E. 2010. The endocranial anatomy of the early sarcopterygian Powichthys from Spitsbergen, based on CT scanning. In: Elliott D.K., Maisey J.G., Yu X.-B., Miao D.-S., editors. Morphology, phylogeny and paleobiogeography of fossil fishes. München: Verlag Dr. Friedrich Pfeil. p. 363-377.

Clément G., Janvier P. 2004. *Powichthys spitsbergensis* sp. nov., a new member of the Dipnomorpha (Sarcopterygii, lobe-finned fishes) from the Lower Devonian of Spitsbergen, with remarks on basal dipnomorph anatomy. Fossils & Strata 50:92-112.

Cloutier R. 1996. The primitive actinistian *Miguashaia bureaui* Schultze (Sarcopterygii). In: Schultze H.-P., Cloutier R., editors. Devonian fishes and plants of Miguasha, Quebec, Canada. München: Verlag Dr. Freidrich Pfeil. p. 227-247.

Cloutier R., Ahlberg P.E. 1996. Morphology, characters, and the interrelationships of basal sarcopterygians. In: Stiasnny M.L.J., Parenti L.R., Johnson G.D., editors. Interrelationships of fishes. San Diego: Academic Press. p. 445- 479.

Coates M.I., Davis S. 2010. About the ears: *Acanthodes* re-examined and gnathostome origin re-analyzed. J. Vert. Paleo. 30 (suppl):74A.

Coates M.I., Gess R.W. 2007. A new reconstruction of Onychoselache Traquairi, comments on early chondrichthyan pectoral girdles and hybodontiform phylogeny. Palaeontology 50:1421-1446.

Coates M.I., Sequeira S.E.K. 1998. The braincase of a primitive shark. Trans. Roy. Soc. Edinb. Earth Sci. 89:63-85.

Coates M.I., Sequeira S.E.K. 2001a. Early sharks and primitive gnathostome interrelationships. In: Ahlberg P.E., editor. Major events in early vertebrate evolution: palaeontology, phylogeny, genetics and development. London: Taylor & Francis. p. 241-262.

Coates M.I., Sequeira S.E.K. 2001b. A new stethacanthid chondrichthyan from the Lower Carboniferous of Bearsden, Scotland. J. Vert. Paleo. 21:438-459.

Coates M.I., Sequeira S.E.K., Sansom I.J., Smith M.M. 1998. Spines and tissues of ancient sharks. Nature 396:729-730.

Davis S.P., Finarelli J.A., Coates M.I. 2012. *Acanthodes* and shark-like conditions in the last common ancestor of modern gnathostomes. Nature 486:247-250.

Denison R.H. 1978. Placodermi. Stuttgart: Gustav Fischer Verlag.

Denison R.H. 1979. Acanthodii. Stuttgart, Gustav Fischer Verlag.

Dennis-Bryan K. 1987. A new species of eastmanosteid arthrodire (Pisces: Placodermi) from Gogo, Western Australia. Zool. J. Linn. Soc. 90:1-64.

Dennis K., Miles R.S. 1981. A pachyosteomorph arthrodire from Gogo, Western Australia. Zool. J. Linn. Soc. 73:213 ~ 258.

Dick J.R.F., Maisey J.G. 1980. The Scottish Lower Carboniferous shark *Onychoselache traquairi*. Palaeontology 23:363-374.

Downs J.P., Donoghue P.C.J. 2009. Skeletal histology of *Bothriolepis canadensis* (Placodermi, Antiarchi) and evolution of the skeleton at the origin of jawed vertebrates. J. Morp. 270:1364-1380.

Dupret V. 2010. Revision of the genus *Kujdanowiaspis* Stensiö, 1942 (Placodermi, Arthrodira, “Actinolepida”) from the Lower Devonian of Podolia (Ukraine). Geodiversitas 32:5-63.

Dupret V., Sanchez S., Goujet D., Tafforeau P., Ahlberg P.E. 2014. A primitive placoderm sheds light on the origin of the jawed vertebrate face. Nature 507:500-503.

Dupret V., Zhu M. 2008. The earliest phyllolepid (Placodermi, Arthrodira) from the Late Lochkovian (Early Devonian) of Yunnan (South China). Geo. Mag. 145:257-278.

Forey P.L. 1998. History of the coelacanth fishes. London: Chapman & Hall.

Forey P.L., Ahlberg P.E., Luksevics E., Zupins I. 2000. A new coelacanth from the Middle Devonian of Latvia. J. Vert. Paleo. 20:243-252.

Gagnier P.-Y. 1996. Acanthodii. In: Schultze H.-P., Cloutier R., editors. Devonian fishes and plants of Miguasha, Quebec, Canada. München: Verlag Dr. Friedrich Pfeil. p. 149-164.

Gagnier P.Y. 1995. Ordovician vertebrates and Agnathan phylogeny. Bull. Mus. Nat. His. Nat. (section C) 17:1-37.

Gagnier P.Y., Hanke G.F., Wilson M.V.H. 1999. *Tetanopsyrus lindoei* gen. et sp. nov., an Early Devonian acanthodian from the Northwest Territories, Canada. Acta Geo. Polon. 49:81-96.

Gagnier P.Y., Wilson M.V.H. 1996a. Early Devonian acanthodians from northern Canada. Palaeontology 39:241-258.

Gagnier P.Y., Wilson M.V.H. 1996b. An unusual acanthodian from northern Canada: revision of Brochoadmones milesi. Mod. Geo. 20:235-251.

Gai Z.-K., Donoghue P.C.J., Zhu M., Janvier P., Stampanoni M. 2011. Fossil jawless fish from China foreshadows early jawed vertebrate anatomy. Nature 476:324-327.

Gardiner B.G. 1984. The relationships of the palaeoniscid fishes, a review based on new specimens of *Mimia* and *Moythomasia* from the Upper Devonian of Western Australia. Bull. Brit. Mus. (Nat. Hist.) Geol. 37:173-428.

Gardiner B.G., Miles R.S. 1994. Eubrachythoracid arthrodires from Gogo, Western Australia. Zool. J. Linn. Soc. 112:443-477.

Giles S., Friedman M., Brazeau M.D. 2015. Osteichthyan-like cranial conditions in an Early Devonian stem gnathostome. Nature 520: 82-85.

Giles S., Rücklin M., Donoghue P.C.J. 2013. Histology of “placoderm” dermal skeletons: Implications for the nature of the ancestral gnathostome. J. Morp. 274:627-644.

Goujet D.F. 1973. *Sigaspis*, un nouvel arthrodire du Dévonien inférieur du Spitsberg. Palaeontogr. Abt. A 143:73-88.

Goujet D.F. 1975. *Dicksonosteus*, un nouvel arthrodire du Dévonien du Spitsberg remarques sur le squelette visceral des Dolichothoraci. In: Lehman J.P., editor. Problèmes actuels de paléontologie-evolution des vertébrés. Paris: Colloques Internationaux du Centre National de la Recherche Scientifique. p. 81-99.

Goujet D.F. 1984. Les poissons placodermes du Spitsberg: Arthrodires Dolichothoraci de la Formation de Wood Bay (Dévonien inférieur). Paris Editions Centre National Recherche Scientifique: Cahiers de Paléontologie.

Goujet D.F., Young G.C. 2004. Placoderm anatomy and phylogeny: new insights. In: Arratia G., Wilson M.V.H., Cloutier R., editors. Recent advances in the origin and early radiation of vertebrates. München: Verlag Dr. Friedrich Pfeil. p. 109-126.

Grogan E.D., Lund R. 2000. *Debeerius ellefseni* (Fam. nov., gen. nov., spec. nov.), an autodiastylic chondrichthyan from the Mississippian bear gulch limestone of Montana (USA), the relationships of the chondrichthyes, and comments on gnathostome evolution. J. Morp. 243:219-245.

Gross W. 1935. Histologische studien am aussenskelett fossiler Agnathen und Fische. Palaeontogr. Abt. A 83:1-60.

Gross W. 1937. Das Kopfskelett von *Cladodus wildungensis*, 1. Endocranium und Palatoquadratum. Senckenbergiana 19:80-107.

Gross W. 1938. Das Kopfskelett von *Cladodus wildungensis* Jaekel. 2, Teil: Der Kieferbogen. Anhang: *Protacrodus vetustus* Jaekel. Senckenbergiana 20:123-145.

Gross W. 1961. *Lunaspis broilii* und *Lunaspis heroldi* aus dem Hunsrückschiefer (Unterdevon, Rheinland). Notiz. Hess. Land. Boden. 89:17-43.

Gross W. 1963. *Gemuendina stuertzi* Traquair. Neuuntersuchung. Notiz. Hess. Land. Boden. 91:36-73.

Gross W. 1969. *Lophosteus superbus* Pander, ein Teleostome aus dem Silur Oesels. Lethaia 2:15-47.

Gross W. 1971. *Lophosteus superbus* Pander: Zähne, Zahnknochen und besondere Schuppenformen. Lethaia 4:131-152.

Halstead L.B. 1979. Internal anatomy of the polybranchiaspids (Agnatha, Galeaspida). Nature 282:833-836.

Hanke G.F. 2008. *Promesacanthus eppleri* n. gen., n. sp., a mesacanthid (Acanthodii, Acanthodiformes) from the Lower Devonian of northern Canada. Geodiversitas 30:287-302.

Hanke G.F., Davis S.P. 2008. Redescription of the acanthodian *Gladiobranchus probaton* Bernacsek & Dineley, 1977, and comments on diplacanthid relationships. Geodiversitas 30:303-330.

Hanke G.F., Davis S.P. 2012. A re-examination of *Lupopsyrus pygmaeus* Bernacsek & Dineley, 1977 (Pisces, Acanthodii). Geodiversitas 34:469-487.

Hanke G.F., Davis S.P., Wilson M.V.H. 2001. New species of the acanthodian genus *Tetanopsyrus* from northern Canada, and comments on related taxa. J. Vert. Paleo. 21:740-753.

Hanke G.F., Wilson M.V.H. 2004. New teleostome fishes and acanthodian systematics. In: Arratia G., Wilson M.V.H., Cloutier R., editors. Recent advances in the origin and early radiation of vertebrates. München: Verlag Dr. Friedrich Pfeil. p. 189-216.

Hanke G.F., Wilson M.V.H. 2006. Anatomy of the Early Devonian Acanthodian *Brochoadmones milesi* based on nearly complete body fossils, with comments on the evolution and development of paired fins. J. Vert. Paleo. 26:526-537.

Hanke G.F., Wilson M.V.H. 2010. The putative stem-group chondrichthyans *Kathemacanthus* and *Seretolepis* from the Lower Devonian MOTH locality, Mackenzie Mountains, Canada. In: Elliott D.K., Maisey J.G., Yu X.-B., Miao D.-S., editors. Morphology, phylogeny and paleobiogeography of fossil fishes. Miinchen: Verlag Dr. Friedrich Pfeil. p. 159-182.

Heidtke U. 1982. Der Xenacanthidae *Orthacanthus senckenbergianus* aus dem pfälzischen Rotliegended (Unter-Perm). Polichia 70:65-86.

Heidtke U. 1998. Revision der Gattung *Orthacanthus* AGASSIZ 1843 (Chondrichthyes: Xenacanthida). Paläontol. Zeit. 72:135-147.

Heintz A. 1937. Die Downtonischen und Devonischen Vertebraten von Spitzbergen VI. *Lunaspis* arten aus dem Devon Spitzbergens. Skr. Sval. Ish. 72:1-23.

Hemmings S.K. 1978. The Old Red Sandstone antiarchs of Scotland: Pterichthyodes and Microbrachius. Monogr. Palaeontogr. Soc. 131:1-64.

Hermus C.R. 2003. Taxonomy and ontogeny of *Ischnacanthus* (Pisces: Acanthodii: Ischnacanthiformes) from the Lower Devonian (Lochkovian), Northwest Territories, Canada. Department of Biological Sciences. Edmonton: University of Alberta.

Holland T., Long J.A. 2009. On the phylogenetic position of *Gogonasus andrewsae* Long 1985, within the Tetrapodomorpha. Acta Zool. 90:285-296.

Janvier P. 1981. The phylogeny of the Craniata, with particular reference to the significance of fossil "agnathans". J. Vert. Paleo. 1:121-159.

Janvier P. 1985. Les Céphalaspides du Spitsberg: anatomie, phylogénie et systématique des Ostéostracés siluro-dévoniens; revisions des Ostéostracés de la Formation de Wood Bay (Dévonien inférieur du Spitsberg). Paris: Cahiers de Paléontologie, Centre national de la Recherche scientifique.

Janvier P. 1996. Early Vertebrates. Oxford: Clarendon Press.

Janvier P., Arsenault M., Desbiens S. 2004. Calcified cartilage in the paired fins of the osteostracan *Escuminaspis laticeps* (Traquair 1880), from the Late Devonian of Miguasha (Québec, Canada), with a consideration of the early evolution of the pectoral fin endoskeleton in vertebrates. J. Vert. Paleo. 24:773-779.

Jarvik E. 1948. Note on the Upper Devonian vertebrate fauna of East Greenland and on the age of the ichthyostegid stegocephalians. Arki. Zool. 41:1-8.

Jarvik E. 1972. Middle and Upper Devonian Porolepiformes from East Greenland with special reference to *Glyptolepis groenlandica* n. sp., and a discussion on the structure of the head in the Porolepiformes. Medd. Grøn. 187:1-307.

Jarvik E. 1977. The systematic position of acanthodian fishes. In: Andrews S.M., Miles R.S., Walker A.D., editors. Problems in vertebrate evolution. London: Academic Press. p. 199-225.

Jarvik E. 1980. Basic structure and evolution of vertebrates, Vol. 1. London: Academic Press.

Jessen H.L. 1966. Die Crossopterygier des Oberen Plattenkalkes (Devon) der Bergisch-Gladbach-Paffrather Mulde (Rheinisches Schiefergebirge) unter Berücksichtigung von amerikanischem und europäischem *Onychodus*-material. Ark. Zool. 18:305-389.

Jessen H.L. 1975. A new choanate fish, *Powichthys thorsteinssoni* n.g., n.sp., from the early Lower Devonian of the Canadian Arctic Archipelago. In: Lehman J.P., editor. Problèmes actuels de paléontologie-evolution des Vertébrés. Paris: Colloques Internationaux du Centre National de la Recherche Scientifique. p. 213-222.

Jessen H.L. 1980. Lower Devonian Porolepiformes from the Canadian Arctic with special reference to *Powichthys thorsteinssoni* Jessen. Palaeontogr. Abt. A 167:180-214.

Johanson Z. 1997. New *Remigolepis* (Placodermi; Antiarchi) from Canowindra, New South Wales, Australia. Geo. Mag. 134:813-846.

Johanson Z., Smith M.M. 2005. Origin and evolution of gnathostome dentitions: a question of teeth and pharyngeal denticles in placoderms. Biol. Rev. 80:303-345.

Liu T.-S., P'an K. 1958. Devonian fishes from Wutung Series near Nanking, China. Palaeontol. Sin., new series C 141:1-41.

Liu Y.-H. 1973. On the new forms of Polybranchiaspiformes and Petalichthyida from Devonian of South - West China. Vert. PalA. 11:132-143.

Liu Y.-H. 1991. On a new petalichthyid, *Eurycaraspis incilis* gen. et sp. nov., from the Middle Devonian of Zhanyi, Yunnan. In: Chang M.-M., Liu Y.-H., Zhang G.-R., editors. Early vertebrates and related problems of evolutionary biology. Beijing: Science Press. p. 139-177.

Long J.A. 1983. A new diplacanthoid acanthodian from the Late Devonian of Victoria. Mem. Ass. Aust. Palaeontol. 1:51-65.

Long J.A. 1985. A new osteolepidid fish from the Upper Devonian Gogo Formation, Western Australia. Rec. West. Aust. Mus. 12:361-377.

Long J.A. 1988. New palaeoniscoid fishes from the Late Devonian and Early Carboniferous of Victoria. Mem. Ass. Aust. Palaeontol. 7:1-64.

Long J.A. 1997. Ptyctodontid fishes (Vertebrata, Placodermi) from the Late Devonian Gogo Formation, Western Australia, with a revision of the European genus *Ctenurella* Ørvig, 1960. Geodiversitas 19:515-555.

Long J.A. 2001. On the relationships of *Psarolepis* and the onychodontiform fishes. J. Vert. Paleo. 21:815-820.

Long J.A., Barwick R.E., Campbell K.S.W. 1997. Osteology and functional morphology of the osteolepiform fish *Gogonasus andrewsae* Long, 1985, from the Upper Devonian Gogo Formation, Western Australia. Rec. West. Aust. Mus. 53:1-89.

Long J.A., Mark-Kurik E., Johanson Z., Lee M.S., Young G.C., ZHU M., Ahlberg P.E., Newman M., Jones R., Blaauwen J.D., Choo B., Trinajstic K. 2015. Copulation in antiarch placoderms and the origin of gnathostome internal fertilization. Nature 517:196-199.

Long J.A., Mark-Kurik E., Young G.C. 2014. Taxonomic revision of buchanosteoid placoderms (Arthrodira) from the Early Devonian of south-eastern Australia and Arctic Russia. Aust. J. Zool. 62:26.

Long J.A., Trinajstic K., Johanson Z. 2009. Devonian arthrodire embryos and the origin of internal fertilization in vertebrates. Nature 457:1124-1127.

Long J.A., Trinajstic K., Young G.C., Senden T. 2008. Live birth in the Devonian period. Nature 453:650-652.

Long J.A., Young G.C., Holland T., Senden T.J., Fitzgerald E.M.G. 2006. An exceptional Devonian fish from Australia sheds light on tetrapod origins. Nature 444:199-202.

Lu J, Giles S, Friedman M, den Blaauwen JL, Zhu M. The oldest actinopterygian highlights the cryptic early history of the hyperdiverse ray-finned fishes. Current Biology. 2016. http://dx.doi.org/10.1016/j.cub.2016.04.045.

Maisey J.G. 1980. An evaluation of jaw suspension in sharks. Am. Mus. Novit. 2706:1-17.

Maisey J.G. 1989a. *Hamiltonichthys mapesi*, g. & sp. nov. (Chondrichthyes; Elasmobranchii), from the Upper Pennsylvanian of Kansas. Am. Mus. Novit. 2931:1-42.

Maisey J.G. 1989b. Visceral skeleton and musculature of a late Devonian shark. J. Vert. Paleo. 9:174-190.

Maisey J.G. 2001. A primitive chondrichthyan braincase from the Middle Devonian of Bolivia. In: Ahlberg P.E., editor. Major Events in Early Vertebrate Evolution: Palaeontology, Phylogeny, Genetics and Development. London: Taylor & Francis. p. 263-288.

Maisey J.G. 2005. Braincase of the Upper Devonian shark *Cladodoides wildungensis* (Chondrichthyes, Elasmobranchii), with observations on the braincase in early chondrichthyans. Bull. Am. Mus. Nat. His. 288:1-103.

Maisey J.G. 2007. The braincase in Paleozoic symmoriiform and cladoselachian sharks. Bull. Am. Mus. Nat. His. 307:1-122.

Maisey J.G., Miller R., Turner S. 2009. The braincase of the chondrichthyan *Doliodus* from the Lower Devonian Campbellton Formation of New Brunswick, Canada. Acta Zool. 90:109-122.

Miles R.S. 1967. Observations on the ptyctodont fish, Rhamphodopsis Watson. Zool. J. Linn. Soc. 47:99-120.

Miles R.S. 1968. Jaw articulation and suspension in *Acanthodes* and their significance. In: Ørvig T., editor. Current problems of lower vertebrate phylogeney. Stockholm: Almqvist & Wiksell. p. 109-127.

Miles R.S. 1971. The Holonematidae (placoderm fishes), a review based on new specimens of *Holonema* from the Upper Devonian of Western Australia. Phil. Trans. R. Soc. Lond. B 263:101-234.

Miles R.S. 1973a. Articulated acanthodian fishes from the Old Red Sandstone of England, with a review of the structure and evolution of the acanthodian shoulder-girdle. Bull. Bri. Mus. (Nat. His.), Geol. 24:111-213.

Miles R.S. 1973b. Relationships of acanthodians. In: Greenwood P.H., Miles R.S., Patterson C., editors. Interrelationships of fishes. London: Academic Press. p. 63-103.

Miles R.S., Westoll T.S. 1968. The placoderm fish *Coccosteus cuspidatus* Miller ex Agassiz from the Middle Old Red Sandstone of Scotland. Part I. Descriptive morphology. Trans. Roy. Soc. Edinb. Earth Sci. 67:373-476.

Miles R.S., Young G.C. 1977. Placoderm interrelationships reconsidered in the light of new ptyctodontids from Gogo, Western Australia. In: Andrews S.M., Miles R.S., Walker A.D., editors. Problems in vertebrate evolution. London: Academic Press. p. 123-198.

Miller R.F., Cloutier R., Turner S. 2003. The oldest articulated chondrichthyan from the Early Devonian peroid. Nature 425:501-504.

Moy-Thomas J.A. 1935. The structure and affinities of *Chondrenchelys problematica*. Tr. Geol. Mag. 105:391-403.

Moy-Thomas J.A. 1936. On the structure and affinities of the Carboniferous Cochliodont *Helodus simplex*. Geol. Mag. 73:488-503.

Newman M.J., Davidson R.G., Den Blaauwen J.L., Burrow C.J. 2011. The Early Devonian Acanthodian *Euthacanthus gracilis* from the midland valley of Scotland. Scot. J. Geol. 47:101-111.

Ørvig T. 1967a. Phylogeny of tooth tissues: evolution of some calcified tissues in early vertebrates. In: Miles A., editor. Structural and chemical organization of teeth. New York: Academic Press. p. 45-110.

Ørvig T. 1967b. Some new acanthodian material from the lower Devonian of Europe. Zool. J. Linn. Soc. 47:131-153.

Ørvig T. 1975. Description, with special reference to the dermal skeleton, of a new radotinid arthrodire from the Gedinnian of Arctic Canada. In: Lehman J.P., editor. Problèmes actuels de paléontologie-evolution des vertébrés. Paris: Colloques Internationaux du Centre National de la Recherche Scientifique. p. 41-71.

Otto M. 1991. Zur systematischen stellung der Lophosteiden (Obersilur, Pisces inc. sedis). Paläontol. Zeit. 65:345-350.

P'an K., Wang S.-T. 1978. Devonian Agnatha and pisces of South China. In: Symposium on the Devonian system of South China. Beijing: Geological Press. p. 298-333.

Pan J. 1992. New galeaspids (Agnatha) from the Silurian and Devonian of China. Beijing: Geological Publishing House.

Pan J., Huo F.-C., Cao J.-X., Gu Q.-C., Liu S.-Y., Wang J.-Q., Gao L.-D., Liu C. 1987. Continental Devonian system of Ningxia and its biotas. Beijing: Geological Publishing House.

Pan J., Wang S., Liu S., Gu Q., Jia H. 1980. Discovery of Devonian *Bothriolepis* and *Remigolepis* in Ningxia. Acta Geol. Sin. 3:176-184.

Pearson D.M. 1982. Primitive bony fishes, with especial reference to *Cheirolepis* and palaeonisciform actinopterygians. Zool. J. Linn. Soc. 74:35-67.

Pearson D.M., Westoll T.S. 1979. The Devonian actinopterygian *Cheirolepis* Agassiz. Trans. Roy. Soc. Edinb. Earth Sci. 70:337-399.

Pradel A., Maisey J.G., Tafforeau P., Janvier P. 2009. An enigmatic gnathostome vertebrate skull from the Middle Devonian of Bolivia. Acta Zool. 90:123-133.

Qiao T., Zhu M. 2010. Cranial morphology of the Silurian sarcopterygian *Guiyu oneiros* (Gnathostomata: Osteichthyes). Sci. Chi. Earth Sci. 53:1836-1848.

Qu Q.-M., Haitina T., Zhu M., Ahlberg P.E. 2015. New genomic and fossil data illuminate the origin of enamel. Nature 526:108-111.

Qu Q.-M., Zhu M., Li G. 2010. Synchrotron radiation X-ray microtomography reveals the primitive histological architecture of osteichthyan scales. In: Abstracts of third international palaeontological congress. London, IPC3 Congress Organising Committees.

Qu Q.-M., Zhu M., Wang W. 2013. Scales and dermal skeletal histology of an Early bony fish *Psarolepis romeri* and their bearing on the evolution of rhombic scales and hard tissues. PloS one 8: e61485.

Rayner D.H. 1951. On the cranial structure of an early palaeoniscid, *Kentuckia* gen. nov. Trans. Roy. Soc. Edinb. Earth Sci. 62:53-83.

Ritchie A. 1973. *Wuttagoonaspis* gen. nov., an unusual arthrodire from the Devonian of Western New South Wales, Australia. Palaeontogr. Abt. A 143:58-72.

Ritchie A. 1975. *Groenlandaspis* in Antarctica, Australia and Europe. Nature 254:569-573.

Ritchie A. 2005. *Cowralepis*, a new genus of phyllolepid fish (Pisces, Placodermi) from the Late Middle Devonian of New South Wales, Australia. Proc. Linn. Soc. NSW 126:215-259.

Ritchie A., Shitao W., Young G.C., Guorui Z. 1992. The Sinolepidae, a family of antiarchs (placoderm fishes) from the Devonian of South China and eastern Australia. Rec. Aust. Mus. 44:319-370.

Romer A.S. 1964. The braincase of the Paleozoic elasmobranch Tamiobatis. Bull. Mus. Comp. Zool. 131:87-105.

Schaeffer B. 1981. The xenacanth shark neurocranium, with comments on elasmobranch monophyly. Bull. Am. Mus. Nat. His. 169:1-66.

Schultze H.-P. 1968. Palaeoniscoidea-schuppen aus dem Unterdevon Australiens und Kansas und aus dem Mitteldevon Spitzbergens. Bull. Bri. Mus. (Nat. His.), Geo. 16:343-368.

Schultze H.-P. 1973. Crossopterygier mit heterozerker Schwanzflosse aus dem Oberdevon Kanadas, nebst einer Beschreibung von Onychodontida-Resten aus dem Mitteldevon Spaniens und aus dem Karbon der USA. Palaeontogr. Abt. A 143:188-208.

Schultze H.-P. 1992. Early Devonian actinoptergians (Osteichthyes, Pisces) from Siberia. In: Mark-Kurik E., editor. Fossil fishes as living animals. Tallinn: Academy of Sciences of Estonia. p. 233-242.

Schultze H.-P., Cumbaa S.L. 2001. *Dialipina* and the characters of basal actinopterygians. In: Ahlberg P.E., editor. Major events in early vertebrate evolution: palaeontology, phylogeny, genetics and development. London: Taylor & Francis. p. 315-332.

Schultze H.-P., Märss T. 2004. Revisiting *Lophosteus* Pander 1856, a primitive osteichthyan. Acta Univ. Latv. 679:57-78.

Schultze H.-P., Zidek J. 1982. Ein primitiver Acanthodier (Pisces) aus dem Unterdevon Lettlands. Paläontol. Zeit. 56:95-105.

Soler-Gijón R. 1999. Occipital spine of *Orthacanthus* (Xenacanthidae, Elasmobranchii): structure and growth. J. Morph. 242:1-45.

Stensiö E. 1969. Elasmobranchiomorphi Placodermata Arthrodires. In: Piveteau J., editor. Traité de paléontologie. Paris: Masson. p. 71-692.

Stensiö E.A. 1963. Anatomical studies on the arthrodiran head. Part 1. Preface, geological and geographical distribution, the organization of the head in the Dolichothoraci, Coccosteomorphi and Pachyosteomorphi. Taxonomic appendix. Kungliga Svenska Vetenskapsakademiens Handlingar.

Stensio E.A.s. 1925. On the head of the macropetalichthyids with certain remarks on the head of the other arthrodires. Geological Series 4:87-197.

Taverne L. 1997. Osorioichthys marginis, "Paléonisciforme" du Famennien de Belgique et la phylogénie des Actinoptérygiens dévoniens (Pisces). Bull. Instit. Roy. Sci. Nat. Belgique, Sci. Terre 67:57-78.

Traquair R.H. 1888. Notes of the nomenclature of the fishes of the Old Red Sandstone of Great Britain. Geo Mag 5:507-517.

Trinajstic K., Boisvert C., Long J., Maksimenko A., Johanson Z. 2015. Pelvic and reproductive structures in placoderms (stem gnathostomes). Biol. Rev. 90:467-501.

Turner S., Burrow C.J., Warren A. 2005. *Gyracanthides hawkinsi* sp. Nov. (Acanthodii, Gyracanthidae) from the Lower Carboniferous of Queensland, Australia, with a review of gyracanthid Taxa. Palaeontology 48:963-1006.

Valiukevicius J. 1992. First articulated *Poracanthodes* from the Lower Devonian of Severnaya Zemlya. In: Mark-Kurik E., editor. Fossil fishes as living animals. Tallinn: Academy of Sciences of Estonia. p. 193-213.

Wang N.-Z. 1991. Two new Silurian galeaspids (jawless craniates) from Zhejiang Province, China, with a discussion of galeaspid-gnathostome relationships. In: Chang M.-M., Liu Y.-H., Zhang G.-R., editors. Early vertebrates and related problems of evolutionary biology. Beijing: Science Press. p. 41-66.

Warren A., Currie B.P., Burrow C., Turner S. 2000. A redescription and reinterpretation of *Gyracanthides murrayi* Woodward 1906 (Acanthodii, Gyracanthidae) from the Lower Carboniferous of the Mansfield Basin, Victoria, Australia. J. Vert. Paleo. 20:225-242.

Watson D.M.S. 1937. The acanthodian fishes. Phil. Trans. Roy. Soc. Lond. B 228:49-146.

Watson D.M.S. 1938. On Rhamphodopsis, ptyctodont from the Middle Devonian Old Red Sandstone of Scotland. Trans. Roy. Soc. Edinb. Earth Sci. 59:397-410.

White E.I. 1978. The larger arthrodiran fishes from the area of Burrinjuck Dam, N.S.W. Trans. Zool. Soc. Lond. 34:149-262.

White E.I., Toombs H.A. 1972. The buchanosteid arthrodires of Australia. Bull. Bri. Mus. (Nat. Hist.) Geol. 22:379-419.

Williams M.E. 1998. A new specimen of *Tamiobatis vetustus* (Chondrichthyes, Ctenacanthoidea) from the Late Devonian Cleveland Shale of Ohio. J. Vert. Paleo. 18:251-260.

Woodward A.S. 1924. On a hybodont shark (Tristychius) from the Calciferous Sandstone Series of Eskdale (Dumfriesshire). Quat. J. Geol. Soc. 80:338-342.

Woodward A.S., White E.J. 1938. The dermal tubercles of the Upper Devonian shark Cladosclache. Ann. Mag. Nat. His. 11:367-368.

Young G.C. 1978. A new Early Devonian petalichthyid fish from the Taemas/Wee Jasper region of New South Wales. Alcheringa 2:103-116.

Young G.C. 1979. New information on the structure and relationships of *Buchanosteus* (Placodermi: Euarthrodira) from the Early Devonian of New South Wales. Zool. J. Linn. Soc. 66:309-352.

Young G.C. 1980. A new Early Devonian placoderm from New South Wales, Australia, with a discussion of placoderm phylogeny. Palaeontogr. Abt. A 167:10-76.

Young G.C. 1984. Reconstruction of the jaws and braincase in the Devonian placoderm fish *Bothriolepis*. Palaeontology 27:635-661.

Young G.C. 1986. The relationships of placoderm fishes. Zool. J. Linn. Soc. 88:1-57.

Young G.C. 1989. New occurrences of culmacanthid acanthodians (Pisces, Devonian) from Antarctica and southeastern Australia. Proc. Linn. Soc. NSW 111:12-25.

Young G.C., Goujet D. 2003. Devonian fish remains from the Dulcie Sandstone and Cravens Peak Beds, Georgina Basin, central Australia. Rec. West. Aust. Mus. Supp. 65:1-85.

Yu X.-B. 1998. A new porolepiform-like fish, *Psarolepis romeri*, gen. et sp. nov. (Sarcopterygii, Osteichthyes) from the Lower Devonian of Yunnan, China. J. Vert. Paleo. 18:261-274.

Zangerl R., Case G.R. 1976. Cobelodus aculeatus (Cope), an snacanthous shark from Pennsylvanian black shales of North America. Palaeontogr. Abt. A 154:107-157.

Zhang G.-R., Wang J.-Q., Wang N.-Z. 2001. The structure of pectoral fin and tail of Yunnanolepidoidei, with a discussion of the pectoral fin of chuchinolepids. Vert. PalA. 39:1-13.

Zhang M.-M. 1980. Preliminary note on a Lower Devonian antiarch from Yunnan, China. Vert. PalA. 18:179-190.

Zhu M. 1991. New information on Diandongpetalichthys (Placodermi: Petalichthyida). In: Chang M.-M., Liu Y.-H., Zhang G.-R., editors. Early vertebrates and related problems of evolutionary biology. p. 179-194.

Zhu M. 1996. The phylogeny of the Antiarcha (Placodermi, Pisces), with the description of Early Devonian antiarchs from Qujing, Yunnan, China. Bull. Mus. Nat. His. Nat. 18:233-347.

Zhu M., Ahlberg P.E. 2004. The origin of the internal nostril of tetrapods. Nature 432:94-97.

Zhu M., Schultze H.-P. 1997. The oldest sarcopterygian fish. Lethaia 30:293-304.

Zhu M., Schultze H.-P. 2001. Interrelationships of basal osteichthyans. In: Ahlberg P., editor. Major events in early vertebrate evolution: palaeontology, phylogeny, genetics and development. London: Taylor & Francis. p. 289-314.

Zhu M., Wang W., Yu X.-B. 2010. *Meemannia eos*, a basal sarcopterygian fish from the Lower Devonian of China –expanded description and significance. In: Elliott D.K., Maisey J.G., Yu X.-B., Miao D.-S., editors. Morphology, phylogeny and paleobiogeography of fossil fishes. München: Verlag Dr. Friedrich Pfeil. p. 199-214.

Zhu M., Yu X.-B. 2002. A primitive fish close to the common ancestor of tetrapods and lungfish. Nature 418:767-770.

Zhu M., Yu X.-B. 2004. Lower jaw character transitions among major sarcopterygian groups - a survey based on new materials from Yunnan, China. In: Arratia G., Wilson M.V.H., Cloutier R., editors. Recent advances in the origin and early radiation of vertebrates. München: Verlag Dr. Friedrich Pfeil. p. 271-286.

Zhu M., Yu X.-B. 2009. Stem sarcopterygians have primitive polybasal fin articulation. Biol. Lett. 5:372-375.

Zhu M., Yu X.-B., Ahlberg P.E. 2001. A primitive sarcopterygian fish with an eyestalk. Nature 410:81-84.

Zhu M., Yu X.-B., Choo B., Qu Q.-M., Jia L.-T., Zhao W.-J., Qiao T., Lu J. 2012a. Fossil fishes from China provide first evidence of dermal pelvic girdles in osteichthyans. PloS one 7:e35103.

Zhu M., Yu X.-B., Janvier P. 1999. A primitive fossil fish sheds light on the origin of bony fishes. Nature 397:607-610.

Zhu M., Yu X.-B., Wang W., Zhao W.-J., Jia L.-T. 2006. A primitive fish provides key characters bearing on deep osteichthyan phylogeny. Nature 441:77-80.

Zhu M., Yu X., Ahlberg P.E., Choo B., Lu J., Qiao T., Qu Q., Zhao W., Jia L., Blom H., Zhu Y.a. 2013. A Silurian placoderm with osteichthyan-like marginal jaw bones. Nature 502:188-193.

Zhu M., Yu X., Choo B., Wang J., Jia L. 2012b. An antiarch placoderm shows that pelvic girdles arose at the root of jawed vertebrates. Biol. Lett. 8:453-456.

Zhu M., Zhao W.-J., Jia L.-T., Lu J., Qiao T., Qu Q.-M. 2009. The oldest articulated osteichthyan reveals mosaic gnathostome characters. Nature 458:469-474.
