# Supplementary material for: Early Gnathostome Phylogeny Revisited: Multiple Method Consensus
Source: PLoS One. 2016 Sep 20;11(9):e0163157. doi: 10.1371/journal.pone.0163157 (PMC5029804; doi:10.1371/journal.pone.0163157)
Supplement: S3 Fig — (PDF) [file pone.0163157.s007.pdf]

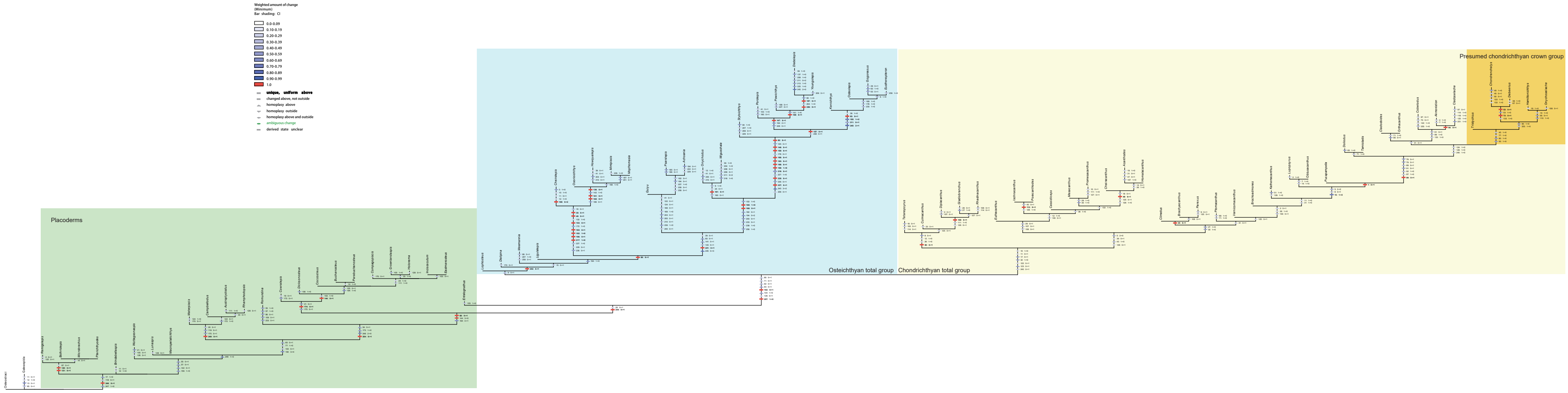

Figure S3. Tracing of characters changing unambiguously on the branches as obtained from MacClade on one of the most parsimonious trees using the revised original dataset.
